# On Creating Deeper Relationship Bonds: Felt Understanding Enhances Relationship Identification

**DOI:** 10.1177/01461672241233419

**Published:** 2024-03-13

**Authors:** Emilie Auger, Sabrina Thai, Carolyn Birnie-Porter, John E. Lydon

**Affiliations:** 1 McGill University, Montreal, Quebec, Canada; 2 Collège Ahuntsic, Montreal, Quebec, Canada; 3 Brock University, St. Catharines, Ontario, Canada; 4 Saint Mary’s University, Halifax, Nova Scotia, Canada

**Keywords:** identity, interpersonal relationships, felt understanding, coherence, meaning-in-life, ease-of-retrieval

## Abstract

Relational experiences play a critical role in shaping how individuals see themselves. In four studies (*N*=945) using person-perception, longitudinal, and experimental designs, we demonstrate that feeling understood changes individuals’ self-concept by increasing the centrality of a specific relationship (relationship identification). Study 1 showed that participants perceived an individual to be more identified with their relationship when their partner was high (vs. low) in understanding. Study 2 extended these results by examining individuals in romantic relationships longitudinally. The results of Studies 1 and 2 were distinct for understanding compared to acceptance and caring. Studies 3 and 4 manipulated felt understanding. Recalling many versus few understanding instances (Study 3) and imagining a close other being low versus high in understanding (Study 4) led individuals to feel less understood, which reduced identification in their friendships and romantic relationships. Furthermore, Study 4 suggests that coherence may be one mechanism through which felt understanding increases relationship identification.

Every story matters . . . We are all worthy of telling our stories and having them heard. We all need to be seen and honored in the same way that we all need to breathe.

Viola Davis (as cited in Brown, 2017)

In an interview, the Oscar-winning actress Viola Davis reveals how feeling understood can be a transformative experience. These experiences are associated with maintaining social relationships (Reis et al., 2017) and increased well-being in daily life (Lun et al., 2008). Furthermore, feeling understood may also transform how individuals define themselves. In the present research, we examined whether feeling understood by a close other leads individuals to see a relationship as centrally important to who they are (relationship identification; Linardatos & Lydon, 2011).

## Relationship Identification

The self-concept consists of a collection of self-aspects (McConnell, 2011). Each self-aspect reflects a meaningful context in individuals’ lives and will be activated depending on contextual cues (Markus & Wurf, 1987). For example, Jane may have self-aspects that represent herself as a student, figure skater, and Mike’s girlfriend, and her relationship self-aspect will be activated when she is with Mike and when she is thinking about Mike. Within each self-aspect are various attributes such as traits, goals, roles, procedural knowledge, and values (Chen et al., 2006) that guide individuals’ behavior and understanding of ongoing experiences (McConnell, 2011). Jane the girlfriend is caring and motivated to protect her relationship. When she encounters an attractive person while on a date with Mike, she may think that he is flirting with her. In contrast, Jane the student is hardworking and has excellent study habits. When she encounters the same attractive person in the library, she may think that he is asking for help on his assignment.

These self-aspects may also represent specific relationships or groups, leading individuals to experience identification, which consists of two components: the cognitive awareness that they are part of a relationship/group and the emotional significance they attach to being part of the relationship/group (Ellemers & Haslam, 2012). Consequently, individuals may be aware that various self-aspects represent specific relationships or groups, but they may not value them to the same degree. Jane may recognize that she is Canadian, but this is not important to her; thus, she does not identify highly as Canadian. In contrast, when individuals value a specific self-aspect, they experience high identification. In the present research, we were interested in what leads individuals to identify with a specific relationship and see this relationship self-aspect as a central part of their self-concept (highly identified; Linardatos & Lydon, 2011).

When a particular self-aspect is seen as central, it is activated more frequently (McConnell, 2011). As this self-aspect becomes activated more often, it requires less contextual cuing (Markus & Wurf, 1987) and is accessible in various situations. If Jane spends a lot of time with Mike, thinks about him often, and values her relationship, her relationship self-aspect will become more accessible and influence how she thinks and behaves in more situations (Linardatos & Lydon, 2011). Consequently, even when she is in the library studying, she may interpret an attractive student’s behavior as flirting and be motivated to protect her relationship.

Indeed, past research has shown that relationship identification predicts relationship-maintenance responses in the face of various threats. Identification predicts resilience in the face of partner transgressions (Auger et al., 2017). Although high identifiers are more acutely reactive to partner transgressions in daily life than low identifiers, these transgressions are less likely to influence their positive global relationship evaluations. In contrast, low identifiers have less positive global relationship evaluations when they experience more transgressions in daily life. High identifiers are also more likely to engage in compensatory responses when faced with more significant relationship threats such as value dissimilarity (Auger et al., 2016): When they are told that their partner does not share important values, high identifiers bolster relationship quality. Finally, because high identifiers have relationship self-aspects that are highly accessible and well-elaborated, they are more likely to engage in relatively spontaneous relationship maintenance responses (Linardatos & Lydon, 2011). For example, they pay less attention to attractive alternatives. In sum, relationship identification plays a significant role in motivating individuals to protect their relationships in the face of adversity.

Because relationship identification focuses on how a *specific* relationship is represented in the self-concept, it differs from other theorizing regarding the self in close relationships. For example, relational-interdependent self-construal (Cross et al., 2000) describes a general tendency to see relationships with others as central to the self-concept, whereas relationship identification refers to a specific relationship. Other approaches examine the development of a new shared identity that individuals form with their partner (couple identity) and investigate the extent to which couples know who they are as a unit (couple identity clarity; Emery et al., 2021) or whether the proportion of self and partner are equal in this merged identity (couple identity fusion; Walsh & Neff, 2018). Relationship identification, however, describes how a specific relationship is represented in one’s self-concept. Furthermore, theoretical perspectives such as inclusion-of-others-in-the-self (Aron et al., 1991) focus on the overlapping of selves (own and partner) and propose that close others are incorporated into the self-concept, leading individuals to see their partner’s attributes as their own. In contrast, identification captures individuals’ awareness that a specific relationship is central to and influences their self-concept, and the extent to which they value this relationship self-aspect is consistent with identity theory (Burke & Reitzes, 1991).

## Perceived Understanding

Despite its importance for relationship maintenance, little is known about how individuals cultivate relationship identification. Consistent with past theorizing, we hypothesize that individuals’ relational experiences determine the extent to which they will identify with the relationship (Lydon & Linardatos, 2012). More specifically, we propose that feeling understood is a critical experience for fostering identification because it allows individuals to reify important aspects of their self-concept through meaningful social interactions.

When individuals want to be understood, they want others to comprehend how they see themselves and their relationship to the world (Reis et al., 2017).^1^ Their unique perspective is shaped by their meaning framework (George & Park, 2016), which is their mental representation of the world and may include core aspects of the self (e.g., self-views, beliefs, and goals; Swann, 2012). When their experiences are consistent with their meaning framework, they experience a sense of coherence: They perceive that their lives and experiences make sense and that things in their lives are coherent and fit together well (George & Park, 2016). This feeling of coherence arises intrapersonally through the consistency between one’s experiences and one’s meaning framework. When they share their experiences with another person, they will feel understood when they believe that the other person “gets” their perspective and experiences (Reis et al., 2017). Consequently, they may view this interaction with the other person as consistent with their meaning framework, which increases coherence interpersonally. Moreover, because individuals believe the other person understands their experience, they may then infer that the meaning framework that they used to make sense of their experiences is also comprehensible to the other person, providing additional evidence that their meaning framework is consistent and coherent.

This desire to understand and make sense of their lives is so strong that individuals value things, such as their self-views, that fulfill this fundamental need highly (Swann, 2012). Indeed, past research has shown that individuals will go to extreme lengths to confirm and protect their self-views because their self-views fulfill this need for coherence (McGregor et al., 2001; Swann, 2012). Other research suggests that self-aspects that provide a greater sense of coherence will be perceived as more important (Costin & Vignoles, 2022). Thus, to the extent that a relationship partner provides individuals with a sense of coherence, the relationship may be valued more because they feel as though life makes more sense when they are in this relationship with this person. This greater valuation of the relationship leads to greater identification by increasing the emotional significance individuals attach to their relationship self-aspect.

Furthermore, felt understanding may lead to cognitive changes that make the relationship more central to the self-concept, leading individuals to identify more highly. When individuals feel fully understood, they are likely to have self-disclosed many private things to their partner (Reis & Shaver, 1988), revealing different self-aspects. For example, Jane may tell Mike about her family (daughter self-aspect) and her studies (student self-aspect) as well as her goals, wishes, and fears. When the partner understands these various self-aspects, individuals may feel as though these different parts of themselves fit together into a cohesive whole in this relationship, which increases coherence (George & Park, 2016). By sharing these various self-aspects with the partner, the relationship self-aspect becomes linked to these other parts of the self (McConnell, 2011), leading the relationship self-aspect to be activated in more contexts and thus influencing how individuals think and behave in more situations. This increases the cognitive component of identification because individuals may become more aware of how the relationship influences their sense of self in a variety of situations. Moreover, because the relationship self-aspect is connected to multiple parts of the self and thus more accessible, it should be seen as more central and important (McConnell, 2011), which also increases emotional significance and thus identification.

In sum, we propose that understanding increases identification through coherence in two ways. First, individuals come to value the relationship more because they feel as though their life makes more sense and is more coherent when they are in this relationship. Second, the relationship becomes more central to the self-concept as individuals disclose more aspects of themselves to their partner, resulting in more connections between the relationship and other parts of the self-concept. This makes the relationship more accessible in a variety of contexts.

## Feeling Understood Versus Caring and Acceptance

Feeling understood may be a particularly potent relational experience because it fulfills fundamental epistemic needs. More specifically, feeling understood fulfills the need for cognitive consistency, the desire to maintain internal consistency among one’s beliefs and propositions (Gawronski, 2012). Cognitive inconsistencies indicate that individuals’ meaning frameworks may contain errors, such as contradictory beliefs, and the accompanying aversive feeling (dissonance) signals that their beliefs may need revision. When individuals feel misunderstood, they may infer that there are inconsistencies in their meaning framework because their own experiences and their beliefs about how another person perceives their experiences are incongruent. This mismatch may lead individuals to question whether they understand themselves and the world, contributing to lower coherence. Alternatively, individuals may infer that the other person does not “get” them and will avoid this person in the future to maintain their sense of coherence (Swann, 2012).

Feeling understood also fulfills belongingness needs (Baumeister & Leary, 1995). Recent theorizing suggests that inconsistencies between one’s own experiences and others’ perceptions threaten the need to belong (Hillman et al., 2023). When others cannot comprehend how individuals experience themselves or the world, or experience these things differently, individuals may infer that they do not belong. Individuals may also question whether close others actually love their true selves or if they love an image of who they are that is not consistent with core aspects of their self and how they experience the world (Pinel et al., 2006), which may also thwart belongingness.

Consequently, individuals may have difficulties feeling connected to others in the absence of understanding even if other components of responsiveness are present (i.e., acceptance and caring; Reis & Shaver, 1988). Indeed, several studies have found that individuals withdraw from others who accept them but do not understand them. For example, individuals are less committed if their spouse has positive views of them when they have negative self-views (Swann et al., 1992). Individuals are also more attracted to a new acquaintance if the acquaintance provides positive feedback that they believe accurately describes them relative to inaccurate feedback (Reis, 2006). Furthermore, in older mother–daughter dyads where both mothers and daughters engage in mutual caregiving, both members are more satisfied with the care they receive when their caregiver knows them better (Martini et al., 2001). Taken together, these studies suggest that feeling understood is a powerful experience on its own and because other processes, such as caregiving and acceptance, are predicated on it (Reis & Clark, 2013). Thus, felt understanding should make unique contributions to identification relative to caring and acceptance.

## Current Research

In four studies, we examined the importance of felt understanding in fostering relationship identification in romantic relationships (Studies 1-4) and friendships (Studies 3-4). We first examined whether feeling understood uniquely predicted identification while controlling for other components of responsiveness (acceptance and caring) and a related construct, inclusion-of-other-in-self, using a person-perception paradigm (Study 1) and a longitudinal design (Study 2). We then established the causal link between felt understanding and identification using ease-of-retrieval (Study 3) and visualization (Study 4) manipulations. Finally, we tested whether coherence mediated the link between understanding and identification (Study 4). Across studies, we predicted that greater felt understanding would increase relationship identification.

## Study 1

In Study 1, we examined whether feeling understood, relative to feeling cared for, is critical in promoting identification. Whereas feeling understood is the sense that someone knows important and core aspects of the self, feeling cared for is the sense that someone shows concern and tries to be supportive (Reis & Clark, 2013). Although these two experiences tend to co-occur and contribute to relationship well-being, we predicted that feeling understood would be more important in promoting identification because it increases coherence, leading individuals to value their relationship self-aspect more. In contrast, feeling cared for may lead individuals to feel more satisfied with their relationship, but not more identified, especially when understanding is lacking.

To manipulate understanding and caring, we used a person-perception paradigm. In the scenarios, the target Jane describes her partner Mike as understanding but not caring or caring but not understanding. We predicted that participants would rate Jane as more identified if she described Mike as high, rather than low, in understanding.

### Method

#### Participants

We recruited 264 participants through CloudResearch Connect to participate in a study on impressions of interpersonal relationships in exchange for $1.50. Our analyses included 261 participants (124 men, 136 women, 1 gender fluid; *M*_age_*=*39.14, *SD*=11.37) who met the inclusion criteria (see Table 1 for exclusions for all studies). In total, 166 were in a relationship. Sensitivity analyses conducted using G*Power (Faul et al., 2007) revealed that with our sample, we could detect an effect of *d*=0.35 with 80% power. We also pre-registered this study (https://osf.io/fx8s6/).^2^ Gender and relationship status effects for all studies are reported in Supplemental Materials (SOM). Study materials, data, and syntax files are available online (https://osf.io/5zmkf/).

**Table 1.** Participant Exclusions for All Studies.

Reason for exclusion	Study 1	Study 2	Study 3	Study 4
Failed the attention check or directed question	1	-	27	58
Did not complete manipulation check or dependent measures	2	1	15	28
Broke up with partner before Time 2 (Study 2)	-	27	-	-
Changed relationship status from pretest to posttest	-	-	19	26
Reported inconsistent information (e.g., partner’s/friend’s name were different)	-	-	31	40
Did not follow instructions	-	-	8	55
Reported instances revealing a lack of understanding (Study 3)	-	-	4	-
Reported fewer instances than instructed (Study 3)	-	-	2	-
Provided single word responses (Study 3)	-	-	2	-
Could not think of an event (Study 4)	-	-	-	8
Reported that their partner/friend would understand them in the low-understanding condition	-	-	-	17
Reported that their partner/friend would not understand them in the high-understanding condition	-	-	-	30
Total	3	28	100	207

*Note.* More details about participants who did not follow instructions can also be found in Supplementary Materials (SOM).

#### Procedure

Participants were randomly assigned to read a scenario: one where Jane felt understood but not as cared for (“I feel that Mike has an intuitive understanding of who I am. . . But at times, I feel like he isn’t being supportive.”; *n*=131), or another where Jane felt cared for but not understood (“I feel that Mike cares for me deeply. . . But at times, I feel like he doesn’t understand me.”; *n*=130). For our manipulation check, participants rated how much Jane felt understood and cared for by Mike. Participants then rated how accepted Jane felt, to ensure we did not inadvertently manipulate acceptance, and how satisfied Jane felt, to ensure that both scenarios were perceived as similarly positive. Next, participants indicated the extent to which they perceived Jane to be identified with her relationship using four items adapted from the Specific Relational-Interdependent Self-Construal (S-RISC) Scale (e.g., “To what extent do you think Jane feels Mike is an important part of who she is?”; Linardatos & Lydon, 2011; α=.88) that were the most sensible for evaluating from a third-person perspective. Finally, participants rated how Jane thinks about her relationship using the inclusion-of-other-in-the-self measure (Aron et al., 1992). All items were rated on a seven-point scale (1 = *not at all* to 7 = *very much*).

### Results

#### Manipulation Check

**Figure 1.**
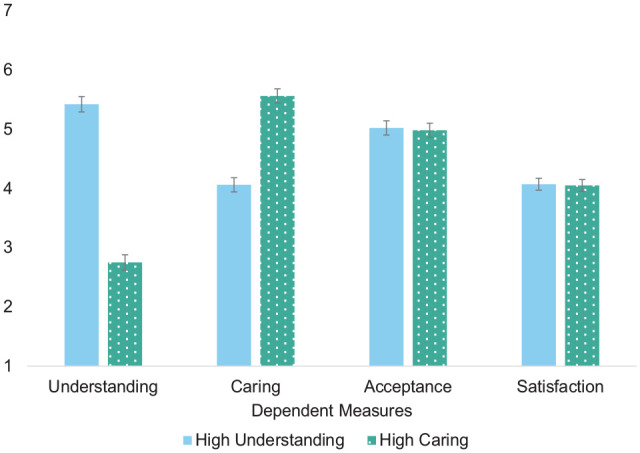
Results From Person-Perception Study (Study 1). *Note.* Error bars represent standard errors.

A mixed 2 × 2 analysis of variance (ANOVA; within: understanding vs. caring rating; between: high understanding vs. high caring) revealed a significant interaction between the ratings and condition, *F*(1, 259)=350.78, *p*<.001, *n*_p_^2^=.58 (Figure 1). Participants perceived Mike to be more understanding in the high-understanding than the high-caring condition, *t*(259)=14.67, *p*<.001. In contrast, they perceived Mike to be more caring in the high-caring than high-understanding condition, *t*(259)=9.06, *p*<.001. The two conditions did not differ in acceptance, *t*(258)=0.18, *p*=.858, nor in satisfaction, *t*(258)=0.10, *p*=.919, suggesting we did not inadvertently manipulate acceptance and that both relationships were viewed as moderately satisfying.

#### Relationship Identification

An independent-samples *t*-test revealed that participants perceived Jane to be more identified in the high-understanding (*M*=4.95, *SE*=0.10) than high-caring (*M*=4.23, *SE*=0.10) condition, *t*(259)=5.04, *p*<.001, *d*=0.62. Next, we examined the extent to which understanding and caring predicted identification using multiple regression (Table 2). Both higher understanding and higher caring predicted greater identification. We then tested whether understanding and caring differed in magnitude (Cohen et al., 2003). Understanding was a larger predictor than caring, *t*(169)=3.58, *p*<.001, suggesting that understanding is a more powerful predictor of identification.

**Table 2.** Multiple Regression Results Predicting Relationship Identification (Studies 1 and 2).

Effect	*b*	*SE*	95% CI		*p*	*r*
			LL	UL		
Model 1 (Study 1)						
Understanding	0.28	0.03	0.20	0.35	<.001	.45
Caring	0.10	0.04	0.01	0.19	.029	.14
Model 2 (Study 1)						
Understanding	0.21	0.04	0.12	0.29	<.001	.32
Caring	-0.01	0.05	-0.12	0.09	.785	.02
Acceptance	0.22	0.06	0.10	0.36	<.001	.22
Model 3 (Study 1)						
IOS	0.48	0.05	0.38	0.59	<.001	.49
Understanding	0.11	0.04	0.03	0.18	.003	.19
Caring	-0.04	0.05	-0.14	0.05	.405	.05
Acceptance	0.12	0.06	0.01	0.24	.026	.14
Time 1 (Study 2)						
IOS	0.30	0.07	0.14	0.49	<.001	.36
Understanding	0.33	0.11	0.03	0.57	.002	.29
Caring	-0.04	0.14	-0.30	0.37	.791	.03
Acceptance	-0.15	0.14	-0.48	0.15	.260	.11
Time 2 (Study 2)						
IOS	0.30	0.07	0.16	0.44	<.001	.37
Understanding	0.33	0.09	0.10	0.58	.001	.32
Caring	0.17	0.14	-0.16	0.46	.221	.12
Acceptance	-0.16	0.13	-0.41	0.13	.228	.11

*Note. r’s* are semi-partial correlations calculated by converting the *t*-statistic. CI = bootstrapped confidence interval with 5,000 resamples; LL = lower limit; UL = upper limit.

Next, we regressed identification onto caring, understanding, and acceptance to determine the unique contributions of each responsiveness component (Table 2).^3^ Understanding and acceptance, but not caring, predicted identification. Taken together, these results suggest that felt understanding is critical for identification after accounting for other facets of responsiveness.

Finally, we sought to demonstrate that although identification was related to inclusion-of-other-in-self, these constructs were distinct and that understanding would predict identification after controlling for inclusion-of-other-in-self. We regressed identification on understanding, acceptance, caring, and inclusion-of-other-in-self simultaneously (Table 2). In this model, inclusion-of-other-in-self, understanding, and acceptance predicted identification, but caring did not. Thus, identification is related to but distinct from inclusion-of-other-in-self, and understanding, but not caring, predicted identification after controlling for inclusion-of-other-in-self.

### Discussion

Overall, these results support our hypothesis that higher understanding, but not higher caring, is associated with higher identification. Participants perceived someone as higher in identification when their partner understood them even if their partner was less caring. Furthermore, although participants perceived similar levels of acceptance in both scenarios, understanding continued to predict identification but caring no longer did when we included acceptance in our model.

We also found that although inclusion-of-other-in-self and identification are related, they are different constructs. Inclusion-of-other-in-self describes how individuals mentally represent *their relationship*, whereas identification describes how individuals represent *their relationship in their self-concept*. Furthermore, because identification reflects a self-aspect and understanding is the sense that someone knows core aspects of the self, understanding continued to predict identification after controlling for inclusion-of-other-in-self, providing further evidence for the unique link between understanding and identification.

Although the manipulation of understanding did not influence acceptance, acceptance also unexpectedly predicted identification; however, it is unclear what participants based their judgments of acceptance on given that the scenarios provided no information about how accepted Jane felt. It is possible that participants based their judgments on their perceptions of other aspects of relationship quality, such as satisfaction. Indeed, acceptance was highly correlated with satisfaction (*r*=.55) relative to the other measures (*r*s<.46). Alternatively, they may have relied on their lay conceptions. Although implicit lay theories are important reflections of one’s relationship experiences and provide normative expectations that may guide future relationship experiences, they are not the same as actual relationship experiences. Moreover, individuals’ feelings of acceptance may be based on more subtle cues that others may miss (Schmader & Sedikides, 2018). Consequently, we examined the importance of understanding, acceptance, and caring in fostering identification in individuals’ own relationships using a longitudinal design.

## Study 2

As in Study 1, we tested whether feeling understood uniquely predicted identification after accounting for caring and acceptance; however, this time we examined these associations using individuals’ reports of their own relationship over an 8-month period. Consistent with Study 1, we expected understanding to continue to predict identification when caring and acceptance were also included in the model.^4^ This study also allowed us to test for the temporal precedence of felt understanding and identification.

### Method

#### Participants

At Time 1, we recruited 210 romantically-involved community participants to complete our questionnaire using various listservs. We planned to recruit individuals, not dyads, currently involved in a relationship. When both members of the couple completed the study, only one member was included in the sample to eliminate non-independence in the data. Fourteen participants were excluded based on this criterion (*N*_Time 1_=196) and were selected at random. At Time 2, 145 participants completed the questionnaire; however, only 118 were still in the same relationship.^5^ Given that relationship identification is specific to a relationship, we conducted analyses for individuals who were still in the same relationship only. One participant did not complete our key measures and was excluded. The final sample included 117 participants (86 women, 31 men; *M*_age_=27.77 years, *SD*=7.66; *M*_relationship length_=5.40 years, *SD*=6.19 years). Fifty-nine participants were dating, 55 were engaged/married, and 3 were dating more than one partner. Participants were compensated with a $7 Amazon gift card for completing Time 1 and a $8 Amazon gift card for completing Time 2. Given our sample, we could detect an effect of *r=*.26 with 80% power.

#### Procedure

At Time 1, participants rated the extent to which they felt understood, accepted, and cared for by their partner on a seven-point scale (1=*Not at all*, 7=*A lot*). They also completed the 11-item S-RISC scale (e.g., “My current romantic relationship is an important reflection of who I am”; Linardatos & Lydon, 2011; α_time1_=.91 and α_time2_=.92) on a seven-point scale (1=*Strongly disagree*; 7=*Strongly agree*). Participants also completed the inclusion-of-other-in-self measure from Study 1. Approximately 8 months later, participants completed the same measures.

### Results

#### Over-Time Analyses

We regressed Time 2 identification on Time 1 understanding, controlling for Time 1 caring, acceptance, and identification. Time 1 identification predicted Time 2 identification, *b*=0.65 CI_95%_ [0.51, 0.81], *SE*=0.08, *z*=8.59, *p*<.001, *r*=.62. Time 1 understanding also uniquely predicted Time 2 identification, *b*=0.18 CI_95%_ [0.02, 0.35], *SE*=0.08, *z*=2.13, *p*=.033, *r*=.19 but caring, *b* = -0.02 CI_95%_ [-0.30, 0.23], *SE*=0.14, *z*=-0.12, *p*=.903, *r*=.01, and acceptance, *b*=-0.05 CI_95%_ [-0.31, 0.20], *SE*=0.13, *z*=-0.41, *p*=.679, *r*=.04, did not.

**Figure 2.**
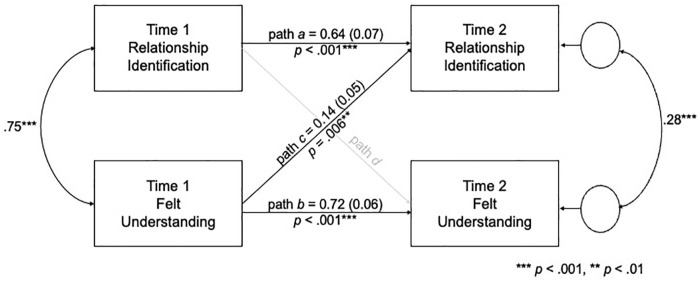
Cross-Lagged Panel Model of the Interrelations Between Relationship Identification and Perceived Understanding (Study 2 Model 2). *Note.* Unstandardized betas and standard errors (in parentheses) are presented. Path *d* was not estimated in this model.

To examine the temporal precedence of understanding and identification, we estimated a cross-lagged panel model (CLPM) analysis. CLPM is useful for testing prospective between-person effects (Orth et al., 2021) and determining temporal predominance between two variables assessed at two different timepoints (Selig & Little, 2012). This model controls for within-person stability in identification and understanding by including autoregressive effects (paths *a* and *b*; Figure 2). It also tests whether the association between Time 1 felt understanding and Time 2 identification (path *c*) is larger than the association between Time 1 identification and Time 2 felt understanding (path *d*). If path *c* is greater than path *d*, understanding is more likely to precede identification in a naturalistic sample than the reverse (Selig & Little, 2012). Moreover, we estimated these models using all available information by implementing full-information maximum likelihood (FIML) estimation (Enders & Bandalos, 2001). Thus, the CLPM was estimated using the full sample.

Model 1 included only the autoregressive effects (paths *a* and *b*). This model did not fit the data well, χ^2^(2)=7.43, *p*=.024, Tucker–Lewis index (TLI) = 0.94, root mean square error of approximation (RMSEA) = 0.12 CI_90%_ [0.04, 0.21], standardized root mean square residual (SRMR) = 0.06. In Model 2 (Figure 2), we added the cross-lagged path between Time 1 understanding and Time 2 identification (path *c*). This model fits the data, χ^2^(1)=0.20, *p*=.657, TLI=1.02, RMSEA=0.00 CI_90%_ [0.00, 0.14], SRMR=0.01, and was significantly better than Model 1, ∆χ^2^(1)=7.23, *p*=.007. In contrast, Model 3, which included the two autoregressive paths (paths *a* and *b*) and the cross-lagged path between Time 1 identification and Time 2 understanding (path *d*), had a relatively poor fit, χ^2^(1)=6.43, *p*=.011, TLI=0.87, RMSEA=0.17 CI_90%_ [0.06, 0.30], SRMR=0.05. Moreover, this cross-lagged path was not significant, *d*=0.09, *SE*=0.09, *p*=.318, and Model 3 did not fit the data better than Model 1, ∆χ^2^(1)=1.00, *p*=.317. Thus, these results support our hypothesis that understanding precedes identification.

#### Inclusion-of-Other-in-Self Versus Identification

As in Study 1, we conducted additional analyses to demonstrate that identification is distinct from inclusion-of-other-in-self and understanding predicts identification after controlling for inclusion-of-other-in-self. We regressed identification on understanding, inclusion-of-other-in-self, acceptance, and caring at each timepoint (Table 2). At Time 1, understanding and inclusion-of-other-in-self predicted identification but not caring or acceptance. We also replicated these findings at Time 2.

### Discussion

Study 2 provided further support that the more participants felt understood by their partner, the more they identified with their romantic relationship 8 months later. Neither caring nor acceptance uniquely predicted identification over time. Our results from the CLPM also suggest that feeling understood in one’s relationship may precede relationship identification. Furthermore, as in Study 1, understanding predicted identification after controlling for inclusion-of-other-in-self. Taken together, Studies 1 and 2 provide strong evidence that feeling understood uniquely predicts identification. However, neither of these studies provided causal evidence. To directly assess the causal role of feeling understood in promoting relationship identification, we manipulated understanding in Studies 3 and 4.

## Study 3

To demonstrate the causal link between understanding and identification, we manipulated felt understanding using the ease-of-retrieval paradigm: Participants generated instances in which they felt understood. We made the task easy or difficult by manipulating whether participants generated few or many instances. Consistent with past research (Schwarz et al., 1991), we expected that experiencing more difficulty when asked to recall many (vs. few) examples would lead individuals to feel less understood by their close others and thus less identified with the relationship.^6^ We suspected that these effects would be small because making individuals feel less understood may be especially threatening for individuals already high in identification. Thus, we chose to use a pretest-posttest experimental design so that participants acted as their own control to increase statistical power (Van Breukelen, 2006). We also explored whether this effect generalizes beyond romantic relationships by examining friendships as well. Indeed, the need to form positive relationships with friends is prominent in adulthood (Collins & Madsen, 2006), and close friends do influence the self-concept (Thai & Lockwood, 2015, 2024; Thai et al., 2019). Romantically involved participants recalled instances about their partner, and single participants recalled instances about a close friend. For both relationship types, we predicted that individuals who struggled to report instances of understanding would feel less understood and identified.

### Method

#### Participants

MTurk participants were recruited to participate in a pretest-posttest experiment. In total, 545 participants completed the pretest questionnaire and were compensated $0.50. Two days later, 459 participants completed the posttest questionnaire and were compensated $2. Of those, 359 (140 men, 218 women; *M*_age_=38.17, *SD*=12.47) met our inclusion criteria. In total, 260 were in a relationship (*M*_length_=10.92 years, *SD*=10.33 years). Using Monte Carlo simulations with 5,000 resamples, sensitivity analyses revealed we could detect an effect of *r*= .02 with 80% power for both the mixed ANOVA and analysis of covariance (ANCOVA; Lane & Hennes, 2018).

#### Procedure

Participants first completed pretest measures of feeling understood and identification.

For felt understanding, participants rated themselves on a nine-point scale (1=*does not understand me at all* to 9=*completely understands me*) in response to the following statement:

On the scale below, indicate the point which best describes the degree to which your partner/friend understands you. The midpoint, “*understands me*,” represents how much most people feel understood by their partner/friend. The scale gradually increases on the right side for those few who experience complete understanding in their intimate relationships and decreases on the left side for those who experience a lower degree of understanding.

The wording for this item was adapted from an item derived from the Spanier Dyadic Adjustment Scale, which has been shown to have good reliability (Goodwin, 1992). To minimize suspicion, participants then completed three S-RISC items (α=.91) that showed the highest loadings in a similar study.

Two days later, participants completed the posttest questionnaire that included our ease-of-retrieval manipulation. Participants thought about situations when they felt understood by their partner/friend in the past month. We instructed them to think about times when their partner/friend understood who they truly are, their thoughts and feelings, and other things that are important to them. Participants were randomly assigned to list either three (easy; *n*=175) or nine instances (difficult; *n*=184). Participants then indicated how difficult it was to generate instances in which they felt understood (1=*not at all difficult*; 10*=very difficult*; Schwarz et al., 1991). This item was recoded such that higher scores reflect greater subjective ease. Finally, participants completed the same understanding item used in the pretest and the full S-RISC scale used in Study 2 (α=.91).

### Results

#### Overview

We conducted analyses in two steps. First, we tested the direct effects of the recall condition on subjective ease, understanding, and identification. Second, because the ease-of-retrieval effect is often mediated by subjective ease (Weingarten & Hutchinson, 2018), we explored whether the recall condition indirectly influenced identification through subjective ease and our proposed intervening mechanism understanding. This mediation model is consistent with Weingarten and Hutchinson’s proposition that to increase the likelihood of detecting an effect between the ease-of-retrieval task and an unrelated dependent measure, researchers should include subjective ease and any additional hypothesized mediators of the ease-of-retrieval manipulation (i.e., understanding). Although we had the same predictions for friendships and romantic relationships, we examined whether the associations differed between relationship types.

#### Direct Effects

We regressed subjective ease on recall condition, relationship type, and their interaction (Table 3). Participants found the task easier if they recalled three (*M*=5.88, standard error [*SE*] = 0.24) rather than nine instances (*M*=3.72, *SE*=0.23). Unexpectedly, participants rated the task as easier in the partner (*M*=5.28, *SE*=0.17) than the friend condition (*M*=4.32, *SE*=0.28). The interaction, however, was not significant.^7^

**Table 3.** Multiple Regression Results for Direct Effects (Study 3).

Effect	*b*	*SE*	95% CI		*p*	*r*
			LL	UL		
Model 1 (Subjective Ease)						
Recall Condition	1.08	0.17	0.76	1.40	<.001	.33
Relationship Type	0.48	0.17	0.16	0.81	.004	.15
Recall × Relationship	-0.29	0.17	-0.60	0.03	.082	.09
Model 2 (Felt Understanding)						
Baseline Understanding	0.70	0.04	0.61	0.79	<.001	.71
Recall Condition	0.16	0.07	0.01	0.30	.019	.13
Relationship Type	-0.36	0.07	-0.51	-0.21	<.001	.29
Recall × Relationship	-0.16	0.07	-0.30	0.00	.021	.13
Simple effect of recall condition for friend	0.31	0.11	0.04	0.57	.006	.15
Simple effect of recall condition for partner	0.002	0.07	-0.13	0.13	.973	.00
Model 3 (Identification)						
Baseline Identification	0.68	0.03	0.60	0.77	<.001	.76
Recall Condition	0.05	0.04	-0.04	0.13	.232	.06
Relationship Type	0.10	0.04	0.02	0.18	.011	.13
Recall × Relationship	-0.11	0.04	-0.19	-0.02	.007	.14
Simple effect of recall condition for friend	0.15	0.07	0.00	0.31	.023	.12
Simple effect of recall condition for partner	-0.06	0.04	-0.13	0.01	.153	.08

*Note.* Recall condition was coded as +1=easy; −1=difficult, and relationship type was coded as −1=friend; +1=partner. *r’s* are semi-partial correlations calculated by converting the *t*-statistic. CI = bootstrapped confidence interval with 5,000 resamples; LL = lower limit; UL = upper limit.

To determine group differences in understanding, we regressed understanding on recall condition, relationship type, and their interaction while controlling for baseline understanding (Table 3). Participants felt more understood in the easy than difficult conditions. Unexpectedly, participants also felt more understood in the friend than partner condition. These effects, however, were qualified by a significant recall-by-relationship interaction. In the friend condition, participants felt more understood in the easy (*M*=8.33, *SE*=0.16) than difficult condition (*M*=7.70, *SE*=0.16). In contrast, recall conditions did not differ in the partner condition.

**Figure 3:**
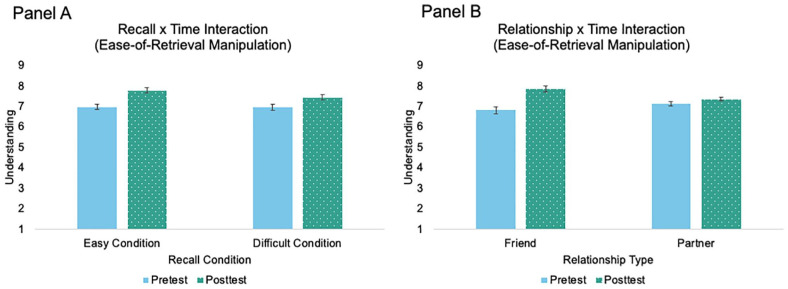
Changes in Felt Understanding From Pretest to Posttest Following Ease-of-Retrieval Manipulation *Note.* Panel A depicts the recall condition x time interaction. Panel B depicts the relationship condition × time interaction. Error bars represent standard errors.

Because we lacked a true control condition, we then examined how understanding changed from pretest to posttest to determine the direction of the manipulation using a 2 × 2 × 2 mixed ANOVA (within: pretest-posttest; between: recall condition, relationship type) and report results regarding the change from pretest to posttest only (Figure 3). There was a significant recall condition-by-time interaction, *F*(1,318)=4.51, *p*=.034, *n*_p_^2^=.01. In both the easy and difficult conditions, understanding increased from pretest to posttest; however, this increase was larger in the easy condition. There was also a significant relationship type-by-time interaction, *F*(1, 318)=30.73, *p*<.001, *n*_p_^2^=.09. In both the friend and partner conditions, understanding increased from pretest to posttest; however, this increase was larger in the friend condition. These results suggest that although we successfully increased understanding in the easy condition, and participants felt more understood in the easy than difficult condition, we inadvertently increased understanding in the difficult condition, albeit to a lesser degree, instead of decreasing it. Moreover, this increase was larger for friends than partners.

We then regressed identification on condition, relationship type, and their interaction while controlling for baseline identification (ANCOVA; Table 3). There was no effect of the recall condition. Participants reported greater identification with their partner than friend condition. These effects, however, were qualified by a significant recall-by-relationship interaction. In the friend condition, participants were more identified in the easy (*M*=5.59, *SE*=0.10) than difficult condition (*M*=5.29, *SE*=0.09). In contrast, recall conditions did not differ in the partner condition.

Taken together, these results suggest that our ease-of-retrieval manipulation influenced subjective ease and was successful in influencing understanding and identification for friends but not partners. Consistent with our hypothesis, participants felt more understood and identified in the easy than difficult condition.

#### Mediational Analysis

Several researchers have highlighted that a manipulation can influence an outcome indirectly even when the direct effect is not significant (Hayes & Rockwood, 2017; Shrout & Bolger, 2002). Thus, the recall condition may have affected the psychological mechanisms by which it was expected to impact identification (i.e., through the indirect effect of recall on subjective ease and understanding). To examine this possibility, we estimated this indirect effect simultaneously for friends and partners. Using a multi-group path analysis and the FIML estimation procedure, we tested whether the indirect effect varied between relationship types (Kline, 2015; Ryu & Cheong, 2017). Consistent with our previous analyses, we included pretest understanding and identification as covariates. A Likelihood Ratio Test revealed that the indirect effect of the recall condition, through subjective ease and felt understanding, on identification did not differ between relationship types, χ^2^(3)=1.54, *p*=.674. Therefore, the indirect paths were estimated using the pooled data. The paths from pretest understanding and pretest identification to subjective ease and the path from recall condition to posttest understanding varied as a function of relationship type and were thus freely estimated. The intercepts and residual variances of subjective ease, posttest understanding, and posttest identification also varied and were also freely estimated.

**Figure 4:**
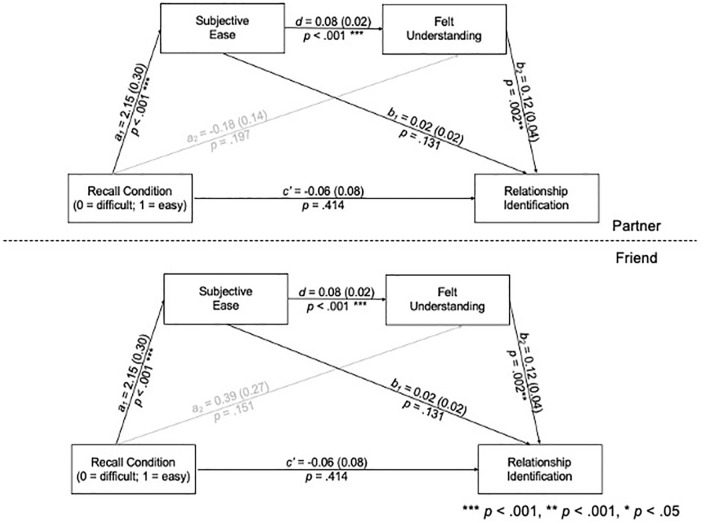
Multi-Group Path Diagram for the Indirect Effect of Recall Condition on Relationship Identification, Through Subjective Ease and Felt Understanding, Controlling for Baseline Felt Understanding and Relationship Identification (Study 3) *Note.* Unstandardized betas and standard errors (in parentheses) are presented. Paths in gray were freely estimated across relationship type.

Participants who generated fewer instances of understanding reported greater subjective ease (see Figure 4 and Table 4), and participants who found it easier to generate examples felt more understood. Participants who felt more understood post-manipulation also reported greater identification, controlling for pretest understanding and identification. The indirect effect of the recall condition, through subjective ease and understanding, on identification, was significant. Although the direct effect of recall condition on understanding (*a*_2_) differed between relationship type, χ^2^(1)=3.91, *p*=.048, neither the path for friends nor partner reached significance, *z*s<1.43, *p*s>.15.

**Table 4:** Summary of Mediation Models From Studies 3 and 4

Study	Experimental condition → mediator 1 (*a_1_* path)	Mediator 1 → mediator 2 (*d* path)	Mediator 2 → identification (*b_2_* path)	Indirect effect (*a_1_db_2_*)
Ease-of-Retrieval Manipulation (Study 3)	*a_1_* = 2.15 (0.30) [1.55, 2.74] *z* = 7.16, *p*<.001***	*d* = 0.08 (0.02) [0.04, 0.13] *z* = 3.49, *p*<.001***	*b_2_* = 0.12 (0.04) [0.04, 0.19] *z* = 3.04, *p* = .002**	*a_1_db_2_* = 0.02 [0.01, 0.05]
Visualization Manipulation (Study 4)	*a_1_* = 0.82 (0.12) [0.58, 1.04] *z* = 6.86, *p*<.001***	*d* = 0.10 (0.04) [0.03, 0.18] *z* = 2.63, *p*<.001***	*b_2_* = 0.13 (0.05) [0.03, 0.22] *z* = 2.67, *p* = .008	*a_1_db_2_* = 0.01 [0.002, 0.03]

*Note.* In Study 3, the experimental condition is the recall condition (easy vs. difficult), Mediator 1 is subjective ease, and Mediator 2 is felt understanding. In Study 4, the experimental condition is a visualization condition (low vs. high understanding), Mediator 1 is felt understanding, and Mediator 2 is coherence. In both studies, all paths reported above did not differ between the partner and friend conditions. Standard errors are reported in parentheses. Bootstrapped 95% confidence intervals with 5,000 resamples are reported in square brackets.

* *p* < .05. **p < .01. ***p < .001.

### Discussion

Study 3 provides further evidence that feeling understood increases identification. When examining the direct effects of the ease-of-retrieval manipulation, we found that individuals felt more understood by a close friend and identified more with that friendship when it was easy, rather than difficult, to think of examples of when they felt understood by their close friend. This was not true for those who thought about their partner. Additional analyses, however, revealed that when we included participants’ reports of subjective ease in our model, the underlying process did not differ for friendships and romantic relationships: The easier it was for individuals to generate moments when they felt understood by their partner/friend, the more they reported feeling understood by this person and in turn more identified with that relationship.

Although the underlying psychological process was similar for both friends and partners, we observed a direct effect of the ease-of-retrieval manipulation for friends but not partners. It is possible that because romantic relationships may be characterized by more intimate interactions, and thus more instances of felt understanding, than friendships, participants in the partner condition may have found it easier to generate examples, regardless of whether they were in the easy or difficult condition. Indeed, participants in the partner condition rated the difficult task as easier than those in the friend condition. The greater subjective ease in the difficult condition resulted in a smaller difference between the recall conditions in the partner than friend condition, which in turn reduced the power of the manipulation to influence felt understanding and identification. Despite the smaller effect in the partner condition, subjective ease still influenced the extent to which participants felt understood and thus identified in the partner condition, suggesting that the process was similar for both friends and partners.

One advantage of the ease-of-retrieval manipulation is that it is subtle. Consequently, individuals are less likely to defend against the threat of *not* feeling understood. Indeed, all participants recalled instances in which they felt understood. This subtle manipulation, however, may have made it more difficult to detect a direct effect of understanding on identification. Moreover, the nature of the manipulation made it difficult to control the content of what participants wrote: Some participants reported instances in which their partner deeply understood their true selves, whereas others reported instances in which their partner understood more trivial things, like what food they were craving. Thus, the experience of the manipulation was heterogeneous across participants, making it more difficult to detect the direct effect. Accordingly, we used a more direct manipulation of understanding in Study 4.

## Study 4

To reduce statistical error due to heterogeneity in our manipulation, we asked participants to imagine their partner/friend being understanding or not after sharing an event that happened in their life that was important to them, but that they had not yet shared with their partner/friend. As in Study 3, romantically-involved participants imagined a scenario with their partner, and single participants imagined a scenario with a close friend. We targeted a specific experience that individuals did not previously share with their close others to make the imagined scenario more plausible and to avoid introducing biases from past experiences with their partner/friend. To further amplify the difference between visualization conditions, we targeted negative experiences. Past research has shown that it can be more difficult to provide responsive support to negative events (Gable et al., 2012). Thus, it may be easier for participants to imagine not feeling understood after sharing a negative event.

Feeling understood in threatening situations, however, may be particularly rewarding. Negative experiences cause greater uncertainty and thus greater disruptions in coherence (Vohs et al., 2019). Consequently, individuals may be more reliant on close others to make sense of these events (King & Hicks, 2021). When a close other can make sense of these events in a way that is consistent with the individual’s meaning framework, individuals may feel even more understood because this ability suggests that the close other truly knows core aspects of the self well. In this situation, the close other plays a critical role in creating coherence for individuals, leading individuals to value this person and their relationship more. As a result, they attach greater emotional significance to their relationship self-aspect. That is, this momentary increase in coherence may lead individuals to identify more strongly with the relationship. Therefore, this manipulation provided the ideal conditions to test our proposed mediation model by examining the indirect effect of visualization conditions on identification through understanding and coherence.

### Method

#### Participants

MTurk participants were recruited to participate in a pretest-posttest experiment. In total, 761 participants completed the pretest questionnaire and received $0.50. One week later, 562 participants completed the manipulation and posttest questionnaire and received $2. Our analyses included 355 participants (147 men, 207 women, 1 transgender; *M*_age_=35.19, *SD*=11.23) who met our inclusion criteria. In total, 234 were in a relationship (*M*_length_=8.42 years, *SD*=6.36 years). Using Monte Carlo simulations with 5,000 resamples, sensitivity analyses revealed we could detect an effect of *r*=.02 with 80% power for both the mixed ANOVA and the ANCOVA.

#### Procedure

Participants completed the same pretest understanding and identification (full scale; α=.93)^8^ measures used in Study 3. Participants also completed five coherence items to measure whether their lives made sense to them (e.g., “My life makes sense”) on a seven-point scale (1=*very strongly disagree*, 7=*very strongly agree*; α=.93; George & Park, 2017).^9^

One week later, participants completed the posttest questionnaire that included our visualization manipulation. We manipulated understanding by modifying Morelli et al.’s (2014) understanding among strangers manipulation. Participants described a negative event that was important to them, but that they had not shared with their partner/friend. They then imagined that they told this person about this event and that this person completely understood their reaction or their thoughts and feelings (*n*=171), or not (*n*=184). To make the manipulation more vivid, participants imagined and then described what the person would say that would make them feel understood or not, and how they would feel. Some negative events participants shared included financial problems, having an extramarital affair, and past abuse.

To further bolster the manipulation, participants indicated their agreement with statements that were compatible with the experimental condition. In the high-understanding condition, the statements were about their partner/friend understanding them (e.g., “My partner/friend knows me better than most people”). In the low-understanding condition, the statements were about their partner/friend not understanding them (e.g., “There are some things that other people know better about me than my partner/friend knows about me”). We used biased anchors (i.e., “*agree at least somewhat”* or “*disagree completely*”; Salancik, 1974), so that participants would endorse most items. Finally, participants completed the same measures of understanding, identification (α=.93), and coherence (α=.95) used in the pretest.

### Results

We conducted analyses in two steps. First, we tested the direct effect of condition on understanding, coherence, and identification. Second, we examined whether the manipulation indirectly influenced identification through a change in understanding and coherence. We expected no differences between partners and friends but tested whether the associations differed.

#### Direct Effects

**Figure 5:**
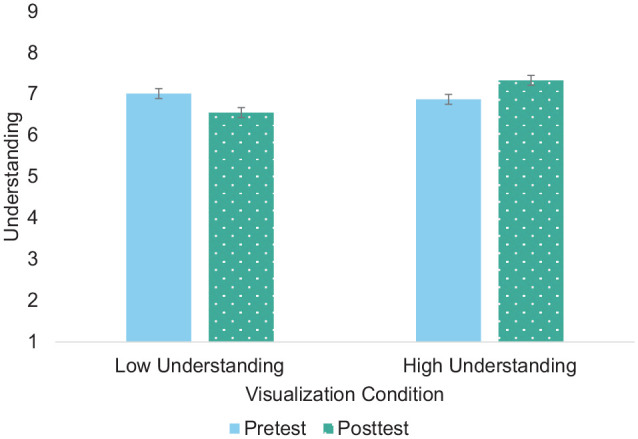
Changes in Felt Understanding From Pretest to Posttest Following Visualization Manipulation *Note.* Error bars represent standard errors.

Because we lacked a true control condition, we first examined how understanding changed from pretest to posttest to determine the direction of the manipulation using a 2 × 2 × 2 mixed ANOVA (within: pretest-posttest; between: visualization, relationship) and report results regarding the change from pretest to posttest only (Figure 5). There was a significant visualization-by-time interaction, *F*(1,350)=40.03, *p*<.001, *n*_p_^2^=.10. In the low-understanding condition, understanding decreased from pretest to posttest, *t*(350)=-4.42, *p*<.001. In the high-understanding condition, understanding increased from pretest to posttest, *t*(350)=4.53, *p*<.001. Thus, our manipulation changed understanding from baseline in the intended directions.

We then regressed felt understanding on visualization condition, relationship type, and their interaction while controlling for baseline understanding (Table 5). Participants felt more understood if they imagined their partner/friend understanding them (*M*=7.42, *SE*=0.09) than not understanding them (*M*=6.56, *SE*=0.09). No other effects were significant.

**Table 5.** Multiple Regression Results for Direct Effects (Study 4).

Effect	*b*	*SE*	95% CI		*p*	*r*
			LL	UL		
Model 1 (Felt Understanding)						
Baseline Understanding	0.61	0.04	0.50	0.72	<.001	.64
Visualization Condition	0.43	0.06	0.31	0.55	<.001	.34
Relationship Type	0.004	0.06	-0.11	0.12	.956	.00
Recall × Relationship	-0.06	0.06	-0.18	0.05	.318	.05
Model 2 (Coherence)						
Baseline Coherence	0.89	0.03	0.83	0.95	<.001	.84
Visualization Condition	0.09	0.04	0.02	0.16	.016	.13
Relationship Type	0.03	0.04	-0.04	0.11	.387	.05
Recall × Relationship	0.02	0.04	-0.06	0.09	.686	.02
Model 3 (Identification)						
Baseline Identification	0.87	0.03	0.82	0.92	<.001	.86
Visualization Condition	0.09	0.03	0.03	0.15	.004	.15
Relationship Type	-0.01	0.03	-0.08	0.06	.783	.01
Recall × Relationship	-0.08	0.03	-0.14	-0.01	.012	.13
Simple effect of condition for friend	0.17	0.05	0.06	0.27	.001	.18
Simple effect of condition for partner	0.01	0.04	-0.06	0.08	.766	.02

*Note.* Visualization condition was coded as -1=low understanding; +1=high understanding, and relationship type was coded as −1=friend; +1=partner. *r’s* are semi-partial correlations calculated by converting the *t*-statistic. CI = bootstrapped confidence interval with 5,000 resamples; LL = lower limit; UL = upper limit.

We then repeated the analysis with coherence as the outcome while controlling for baseline coherence (Table 5). Participants said their lives made more sense if they imagined their partner/friend understanding them (*M*=5.07, *SE*=0.05) than not understanding them (*M*=4.88, *SE*=0.05). No other effects were significant.

Finally, we repeated the analysis with identification as the outcome while controlling for baseline identification (Table 5). Participants felt more identified in the high than low understanding condition. There was no effect of relationship type; however, there was a significant interaction. In the friend condition, participants reported greater identification if they imagined their *friend* understanding them (*M*=5.59, *SE=*0.07) than not understanding them (*M*=5.25, *SE*=0.07). In contrast, visualization conditions did not differ in the partner condition, a result we return to in the General Discussion.

#### Mediational Analysis

We examined whether the visualization manipulation may have affected identification indirectly through understanding and coherence. As in Study 3, we estimated the indirect effect simultaneously for friends and partners. A Likelihood Ratio Test revealed that the indirect effect of visualization condition on identification, through understanding and coherence, did not differ between relationship types, χ^2^(3)=3.86, *p*=.277. Thus, the indirect paths were estimated using the pooled data. Pretest understanding, identification, and coherence were included as covariates. The paths from pretest understanding to posttest understanding, pretest understanding to posttest identification, and pretest identification to posttest identification varied as a function of relationship type and were thus freely estimated.

**Figure 6:**
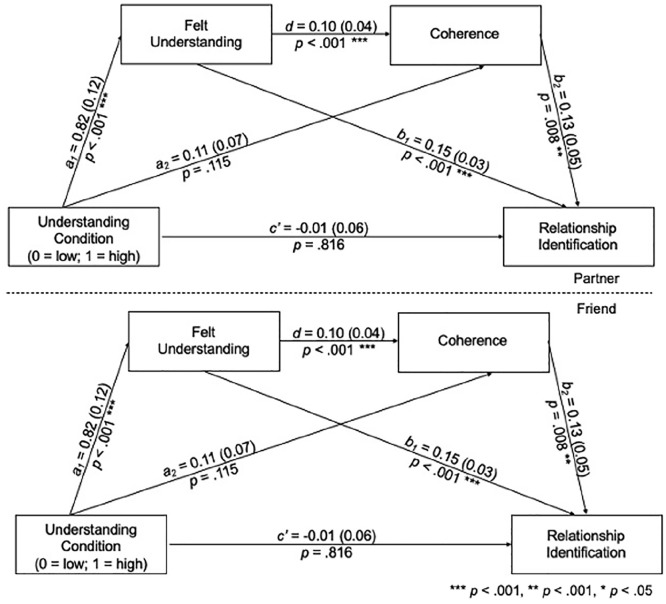
Multi-Group Path Diagram for the Indirect Effect of Understanding Condition on Relationship Identification, Through Feeling Understood and Coherence, Controlling for Baseline Understanding, Relationship Identification, and Coherence (Study 4) *Note.* Unstandardized betas and standard errors (in parentheses) are presented.

Participants who imagined their close other responding in an understanding manner felt more understood after the manipulation (see Figure 6 and Table 4), and participants who felt more understood reported greater coherence. In turn, participants with higher levels of coherence post-manipulation were more identified, controlling for baseline understanding and identification. There was a significant indirect effect of condition on identification, through understanding and coherence.

### Discussion

Our manipulation influenced understanding, coherence, and identification for friendships. Moreover, our mediational analysis replicated and extended Study 3 by revealing coherence as a potential mediator between understanding and identification for both friendships and romantic relationships. Individuals felt more understood if they imagined their close other understanding them than not understanding them, and this led individuals to report greater coherence and identification. Consistent with our theorizing, our results suggest that a sense of coherence may mediate the association between understanding and identification: When individuals feel more understood by a close other, they also feel as though life makes more sense with this person and this, in turn, leads them to identify with that specific relationship more. These results also suggest that feeling understood may buffer individuals from negative experiences: When facing challenging circumstances or adverse events, feeling understood may help individuals gain valuable resources, such as a greater sense of coherence and identification, to cope with the situation.

## General Discussion

Extensive research has shown that relationships are a fundamental aspect of the self-concept (Baldwin, 1992; Chen et al., 2006). The present studies shed light on what relational experiences lead individuals to see a specific relationship as a valued, central part of the self. Across four studies using diverse methods, we found strong and consistent evidence that feeling understood by someone leads individuals to perceive their relationship with this person as central to their self-concept. Although past research has shown that felt understanding promotes relationship quality and personal well-being (Reis et al., 2017), the present research suggests that feeling understood may promote these positive outcomes by changing the self-concept: Individuals value the self-aspects that represent relationships with understanding close others more, elevating their importance in one’s self-concept. This increased importance then promotes various relationship maintenance processes to sustain the relationship in the face of adversity.

The present research also highlights the importance of understanding, relative to other responsiveness components (caring and acceptance). Understanding consistently predicted identification over and above caring, acceptance, and inclusion-of-other-in-self (Studies 1 and 2), suggesting that it plays a significant role in influencing how specific relationships change the self-concept. Unlike caring and acceptance, feeling understood may fulfill individuals’ fundamental need for cognitive consistency, which reassures them their meaning framework is cohesive and consistent and thus useful for organizing and interpreting their experiences. This consistency, in turn, increases the sense that individuals understand themselves well, increasing coherence (Swann et al., 1992), which leads them to value the relationship more. Indeed, Study 4 confirmed that when individuals felt more understood, they also experienced a greater sense of coherence, and this in turn increased their relationship identification. We also replicated our mediation model in Study 1 (see SOM).

Our results also suggest that individuals may identify with a relationship if they feel understood, even when acceptance and care are lacking. In Studies 1 and 2, understanding continued to predict identification when acceptance and caring are held constant, suggesting that more felt understanding will predict greater identification regardless of how caring and accepting the relationship is. Moreover, in an additional person-perception study (see SOM), we found that participants still perceived an individual to be more identified when her relationship was described as high in understanding but much lower in caring than the high-understanding scenario used in Study 1, suggesting that individuals believe that others will identify highly with a relationship even when caring is lacking. One theoretical implication of these findings is that as feeling understood promotes identification, it may be difficult for individuals to disidentify with a relationship lacking in acceptance and caring: The relationship may remain personally important because of the psychological benefits of feeling understood. Thus, our studies provide some clues as to why some individuals may stay in harmful relationships: They may stay because their partner makes them feel understood, increasing coherence and identification. Consequently, it is possible that leaving the relationship would threaten their understanding of themselves and the world. That is, the perceived understanding built with a partner over the course of a relationship is an investment that would be lost if the relationship were to end. Further, when the costs of giving up an identity are high, individuals are especially likely to hold on to that identity (Stryker, 1980). Therefore, feeling understood and strongly identified with a relationship may have a powerful hold over individuals, an issue that should be explored in future research.

Although we successfully manipulated understanding, we measured our mediator coherence, making alternative causal pathways possible. For example, individuals also derive meaning from their identity (McGregor & Little, 1998). Thus, it is possible that developing a strong sense of relationship identification also increases coherence. We note that our proposed causal direction, that understanding leads to greater coherence, which in turn increases identification, is compatible with other theoretical approaches such as self-verification theory (Swann, 2012). Indeed, past research has shown that when their partner self-verifies them (akin to understanding), individuals feel as though they know themselves better (akin to coherence), and this feeling of knowing the self better predicts greater commitment (akin to identification; Swann et al., 1992). Nevertheless, future research should use alternative methodological approaches to determine the causal chain.

We also found that the psychological processes that increase identification in romantic relationships also extend to friendships (Studies 3-4). That is, when individuals feel more understood by their friends, they identify more with their friendship. Despite similarities in the underlying processes, our manipulations caused larger effects for friends than partners. These manipulations may have been more powerful for friends because individuals expect their partners to understand them more than their friends. Indeed, Study 3 supports this possibility by showing that individuals found it easier to generate examples of feeling understood by their partner than by their friend.

It is also possible that these differences between friend and partner would be eliminated if individuals identified with their friend as much as they identified with their partner. In both experimental studies, participants were initially more identified in the partner than friend condition. Consequently, participants may have been motivated to overperceive how understood they felt by their partner to protect their relationship (Reis et al., 2017), resulting in smaller effects. Greater identification in romantic relationships may have also made it more difficult to change identification because that relationship self-aspect is more chronically accessible. Indeed, past research suggests that although chronically accessible constructs are malleable, changes to these constructs are short-lived at best (Lai et al., 2016). Thus, it is possible that a single instance of understanding or misunderstanding is unlikely to lead to lasting changes when individuals are highly identified. Indeed, baseline identification accounted for a substantial portion of the variance in Studies 3 and 4, suggesting that it is highly stable over time. Moreover, these subtle changes may be better captured using cognitive measures than self-reports, a fascinating direction for future research. An alternative possibility is that for high identifiers, instances of misunderstanding may be more impactful than instances of understanding because they have obtained optimal levels of belonging (Hillman et al., 2023). Therefore, more understanding confers smaller rewards but instances of misunderstanding may be particularly aversive. Furthermore, individuals may be adept at defending against one instance of misunderstanding; however, it may be more difficult to engage in these motivated defenses when they consistently feel misunderstood. Future research should examine whether there are cumulative effects of instances of understanding or misunderstanding on identification, especially for high identifiers.

In Study 4, we created a strong manipulation of understanding to demonstrate the causal link; however, this led us to target an experience that participants had not yet shared with their partner/friend. Targeting this specific experience may have systematically influenced our results, limiting the generalizability of our results, because participants may have various reasons for not sharing the experience with their partner/friend. For example, they may have refrained from this disclosure due to potential distress or threat to the partner/friend. We note, however, that we believe that these results should generalize because we replicated the mediation model in Study 1. Moreover, negative experiences that individuals feel comfortable sharing may still lead to the same psychological processes because they will also cause disruptions to existing meaning frameworks, prompting individuals to seek out close others to make sense of them to restore a sense of coherence and in turn increase identification. It is also possible that these effects may be limited to negative but not positive experiences given that individuals are more likely to find meaning following negative than positive events (Vohs et al., 2019). Other research examining shared reality for positive experiences (e.g., taste in music) and interpersonal connection has found that individuals felt more connected to a person after learning that the other person had the same subjective experience as them, which may also boost understanding (Pinel et al., 2006; Rossignac-Milon et al., 2021; Rossignac-Milon & Higgins, 2018). Moreover, these shared positive experiences also fulfill the fundamental need for cognitive consistency, reinforcing a sense of coherence. Nevertheless, additional research will be necessary to examine whether understanding promotes identification through coherence in a variety of experiences.

On a daily basis, individuals encounter numerous opportunities to feel understood by others. The present research suggests that feeling understood is a powerful experience that imbues specific relationships with importance and value, making it more self-defining. When individuals feel understood by a close other, they feel as though the world and themselves make more sense. This experience is so powerful that it leads individuals to value relationships with understanding close others more, elevating its importance in their self-concept.

## Supplemental Material

sj-docx-1-psp-10.1177_01461672241233419 – Supplemental material for On Creating Deeper Relationship Bonds: Felt Understanding Enhances Relationship Identification Supplemental material, sj-docx-1-psp-10.1177_01461672241233419 for On Creating Deeper Relationship Bonds: Felt Understanding Enhances Relationship Identification by Emilie Auger, Sabrina Thai, Carolyn Birnie-Porter and John E. Lydon in Personality and Social Psychology Bulletin

## Notes

1. Because felt understanding is the belief that another person sees themselves and the world in the unique way that they do, it differs from shared reality, which arises when two or more individuals see and experience the *external* world similarly (Rossignac-Milon et al., 2021).

2. Study 1 is a replication of a previous study. The results were mostly consistent across the two studies. See SOM (https://osf.io/5zmkf/) for the original study’s results.

3. For transparency, we have also reported the results of these models with each predictor on its own in the SOM.

4. In Study 2, we found that understanding did not predict satisfaction over time. See SOM for results.

5. Attrition analyses are reported in SOM.

6. To determine the number of instances for each condition, we conducted two validation studies (see SOM).

7. Because the ease-of-retrieval effect differed between relationship types, we explored this interaction to gain a better understanding of the differences between the relationship conditions. There was no difference between the relationship types in the easy condition, *b*=0.19 CI_95%_ [−0.24, 0.66], *SE*=0.24, *t*(354)=0.80, *p*=.424, *r*=.04; however, participants in the partner condition (*M*=4.49, *SE*=0.25) found the difficult condition to be easier than those in the friend condition (*M*=2.94, *SE*=0.38), *b*=0.77, CI_95%_ [0.32, 1.22], *SE*=0.23, *t*(354)=3.41, *p*=.001, *r*=.18. Moreover, the difference between the recall conditions was smaller in the partner condition, *b*=0.79 CI_95%_ [0.44, 1.14], *SE*=0.17, *t*(354)=4.55, *p*<.001, *r*=.23, than in the friend condition, *b*=1.37 CI_95%_ [0.83, 1.90], *SE*=0.28, *t*(354)=4.84, *p*<.001, *r*=.25.

8. Because the two questionnaires were 1 week apart, we used the full scale to increase our ability to detect changes.

9. We also measured the other components of meaning, purpose, and mattering; however, we did not find any effect of condition or felt understanding on mattering and purpose (see SOM).

## References

[bibr1-01461672241233419] Aron A. Aron E. N. Smollan D. (1992). Inclusion of other in the self scale and the structure of interpersonal closeness. Journal of Personality and Social Psychology, 63, 596–612.

[bibr2-01461672241233419] Aron A. Aron E. N. Tudor M. Nelson G. (1991). Close relationships as including other in the self. Journal of Personality and Social Psychology, 60, 241–253.

[bibr3-01461672241233419] Auger E. Hurley S. Lydon J. E. (2016). Compensatory relationship enhancement: An identity motivated response to relationship threat. Social Psychological and Personality Science, 7, 223–231.

[bibr4-01461672241233419] Auger E. Menzies-Toman D. Lydon J. E. (2017). Daily experiences and relationship well-being: The paradoxical effects of relationship identification. Journal of Personality, 85, 741–752. 27589212 10.1111/jopy.12283

[bibr5-01461672241233419] Baldwin M. W. (1992). Relational schemas and the processing of social information. Psychological Bulletin, 112, 461–484.

[bibr6-01461672241233419] Baumeister R. F. Leary M. R. (1995). The need to belong: Desire for interpersonal attachments as a fundamental human motivation. Psychological Bulletin, 117, 497–529. 7777651

[bibr7-01461672241233419] Brown B. (2017). Braving the wilderness: The quest for true belonging and the courage to stand alone. Random House.

[bibr8-01461672241233419] Burke P. J. Reitzes D. C. (1991). An identity theory approach to commitment. Social Psychology Quarterly, 54, 239–251.

[bibr9-01461672241233419] Chen S. Boucher H. C. Tapias M. P. (2006). The relational self revealed: Integrative conceptualization and implications for interpersonal life. Psychological Bulletin, 132, 151–179. 16536640 10.1037/0033-2909.132.2.151

[bibr10-01461672241233419] Cohen J. Cohen P. West S. G. Aiken L. S. (2003). Applied multiple regression/correlation analysis for the behavioral sciences. Routledge.

[bibr11-01461672241233419] Collins W. A. Madsen S. D. (2006). Personal relationships in adolescence and early childhood. In Vangelisti A. L. Perlman D. (Eds.), The Cambridge handbook of personal relationships (pp. 191–210). Cambridge University Press.

[bibr12-01461672241233419] Costin V. Vignoles V. L. (2022). What do people find most meaningful? How representations of the self and the world provide meaning in life. Journal of Personality, 90, 541–558. 34655471 10.1111/jopy.12682

[bibr13-01461672241233419] Cross S. E. Bacon P. L. Morris M. L. (2000). The relational- interdependent self-construal and relationships. Journal of Personality and Social Psychology, 78, 791–808. 10794381

[bibr14-01461672241233419] Ellemers N. Haslam S. A. (2012). Social identity theory. In Van Lange P. Kruglanski A. Higgins E. T. (Eds.), Handbook of theories of social psychology (pp. 379–398). Sage.

[bibr15-01461672241233419] Emery L. F. Gardner W. L. Carswell K. L. Finkel E. J. (2021). Who are “we”? Couple identity clarity and romantic relationship commitment. Personality and Social Psychology Bulletin, 47, 146–160. 32400297 10.1177/0146167220921717

[bibr16-01461672241233419] Enders C. K. Bandalos D. L. (2001). The relative performance of full information maximum likelihood estimation for missing data in structural equation models. Structural Equation Modeling, 8, 430–457.

[bibr17-01461672241233419] Faul F. Erdfelder E. Lang A. G. Buchner A. (2007). G*Power 3: A flexible statistical power analysis program for the social, behavioral, and biomedical sciences. Behavior Research Methods, 39, 175–191. 17695343 10.3758/bf03193146

[bibr18-01461672241233419] Gable S. L. Gosnell C. L. Maisel N. C. Strachman A. (2012). Safely testing the alarm: Close others’ responses to personal positive events. Journal of Personality and Social Psychology, 103, 963–981. 22889071 10.1037/a0029488

[bibr19-01461672241233419] Gawronski B. (2012). Back to the future of dissonance theory: Cognitive consistency as a core motive. Social Cognition, 30, 652–668.

[bibr20-01461672241233419] George L. S. Park C. L. (2016). Meaning in life as comprehension, purpose, and mattering: Toward integration and new research questions. Review of General Psychology, 20, 205–220.

[bibr21-01461672241233419] George L. S. Park C. L. (2017). The multidimensional existential meaning scale: A tripartite approach to measuring meaning in life. The Journal of Positive Psychology, 12, 613–627.

[bibr22-01461672241233419] Goodwin R. (1992). Overall, just how happy are you? The magical question 31 of the Spanier Dyadic Adjustment Scale. Family Therapy: The Journal of the California Graduate School of Family Psychology, 19, 273–275.

[bibr23-01461672241233419] Hayes A. F. Rockwood N. J. (2017). Regression-based statistical mediation and moderation analysis in clinical research: Observations, recommendations, and implementation. Behavior Research and Therapy, 98, 39–57. 10.1016/j.brat.2016.11.001 27865431

[bibr24-01461672241233419] Hillman J. G. Fowlie D. I. MacDonald T. K. (2023). Social verification theory: A new way to conceptualize validation, dissonance, and belonging. Personality and Social Psychology Review, 27, 309–331. 36461780 10.1177/10888683221138384 PMC10363943

[bibr25-01461672241233419] King L. A. Hicks J. A. (2021). The science of meaning in life. Annual Review of Psychology, 72, 561–584. 10.1146/annurev-psych-072420-122921 32898466

[bibr26-01461672241233419] Kline R. B. (2015). Principles and practice of structural equation modeling. Guilford.

[bibr27-01461672241233419] Lai C. K. Skinner A. L. Cooley E. Murrar S. Brauer M. Devos T. Calanchini J. Xiao Y. J. Pedram C. Marshburn C. K. Simon S. Blanchar J. C. Joy-Gaba J. A. Conway J. Redford L. Klein R. A. Roussos G. Schellhaas F. M. H. Burns M. . . . Nosek B. A. (2016). Reducing implicit racial preferences: II. Intervention effectiveness across time. Journal of Experimental Psychology: General, 145, 1001–1016. 27454041 10.1037/xge0000179

[bibr28-01461672241233419] Lane S. P. Hennes E. P. (2018). Power struggles: Estimating sample size for multilevel relationships research. Journal of Social and Personal Relationships, 35, 7–31.

[bibr29-01461672241233419] Linardatos L. Lydon J. E. (2011). Relationship-specific identification and spontaneous relationship maintenance processes. Journal of Personality and Social Psychology, 101, 737–753. 21728451 10.1037/a0023647

[bibr30-01461672241233419] Lun J. Kesebir S. Oishi S. (2008). On feeling understood and feeling well: The role of interdependence. Journal of Research in Personality, 42, 1623–1628. 19956355 10.1016/j.jrp.2008.06.009 PMC2652476

[bibr31-01461672241233419] Lydon J. E. Linardatos L. (2012). Identification: The why of relationship commitment. In Campbell L. LaGuardia J. G. Olson J. M. Zanna M. P. (Eds.), The Ontario symposium on personality and social psychology: Vol. 12. The science of the couple (pp. 119–140). Psychology Press.

[bibr32-01461672241233419] Markus H. Wurf E. (1987). The dynamic self-concept: A social psychological perspective. Annual Review of Psychology, 38, 299–337.

[bibr33-01461672241233419] Martini T. S. Grusec J. E. Bernardini S. C. (2001). Effects of interpersonal control, perspective taking, and attributions on older mothers’ and adult daughters’ satisfaction with their helping relationships. Journal of Family Psychology, 15, 688–705. 11770475 10.1037//0893-3200.15.4.688

[bibr34-01461672241233419] McConnell A. R. (2011). The multiple self-aspects framework: Self-concept representation and its implications. Personality and Social Psychology Review, 15, 3–27. 20539023 10.1177/1088868310371101

[bibr35-01461672241233419] McGregor I. Little B. R. (1998). Personal projects, happiness, and meaning: On doing well and being yourself. Journal of Personality and Social Psychology, 74, 494–512. 9491589 10.1037//0022-3514.74.2.494

[bibr36-01461672241233419] McGregor I. Zanna M. P. Holmes J. G. Spencer S. J. (2001). Compensatory conviction in the face of personal uncertainty: Going to extremes and being oneself. Journal of Personality and Social Psychology, 80, 472–488. 11300580 10.1037/0022-3514.80.3.472

[bibr37-01461672241233419] Morelli S. A. Torre J. B. Eisenberger N. I. (2014). The neural bases of feeling understood and not understood. Social Cognitive and Affective Neuroscience, 9, 1890–1896. 24396002 10.1093/scan/nst191 PMC4249470

[bibr38-01461672241233419] Orth U. Clark D. A. Donnellan M. B. Robins R. W. (2021). Testing prospective effects in longitudinal research: Comparing seven competing cross-lagged models. Journal of Personality and Social Psychology, 120, 1013–1034. 32730068 10.1037/pspp0000358 PMC7854859

[bibr39-01461672241233419] Pinel E. C. Long A. E. Landau M. J. Alexander K. Pyszczynski T. (2006). Seeing I to I: A pathway to interpersonal connectedness. Journal of Personality and Social Psychology, 90, 243–257. 16536649 10.1037/0022-3514.90.2.243 PMC2658264

[bibr40-01461672241233419] Reis H. T. (2006). Implications of attachment theory for research on intimacy. In Mikulincer M. Goodman G. S. (Eds.), Dynamics of romantic love: Attachment, caregiving, and sex (pp. 383–403). Guilford Press.

[bibr41-01461672241233419] Reis H. T. Clark M. S. (2013). Responsiveness. In Simpson J. A. Campbell L. (Eds.), The Oxford handbook of close relationships (pp. 400–423). Oxford University Press.

[bibr42-01461672241233419] Reis H. T. Lemay E. P. Finkenauer C. (2017). Toward understanding understanding: The importance of feeling understood in relationships. Social and Personality Psychology Compass, 11, 1–22.

[bibr43-01461672241233419] Reis H. T. Shaver P. (1988). Intimacy as an interpersonal process. In Duck S.W. (Ed.), Handbook of personal relationships (pp. 376–389). Wiley.

[bibr44-01461672241233419] Rossignac-Milon M. Bolger N. Zee K. Boothby E. Higgins E. T. (2021). Merged minds: Generalized shared reality in dyadic relationships. Journal of Personality and Social Psychology, 120, 882–911. 32673045 10.1037/pspi0000266

[bibr45-01461672241233419] Rossignac-Milon M. Higgins E. T. (2018). Epistemic companions: Shared reality development in close relationships. Current Opinion in Psychology, 23, 66–71. 29360060 10.1016/j.copsyc.2018.01.001

[bibr46-01461672241233419] Ryu E. Cheong J. (2017). Comparing indirect effects in different groups in single-group and multi-group structural equation models. Frontiers in Psychology, 8, Article 747. 10.3389/fpsyg.2017.00747 PMC5425601 28553248

[bibr47-01461672241233419] Salancik J. R. (1974). Inference of one’s attitude from behavior recalled under linguistically manipulated cognitive sets. Journal of Experimental Social Psychology, 10, 415–427.

[bibr48-01461672241233419] Schmader T. Sedikides C. (2018). State authenticity as fit to environment: The implications of social identity for fit, authenticity, and self-segregation. Personality and Social Psychology Review, 22, 228–259. 28975851 10.1177/1088868317734080

[bibr49-01461672241233419] Schwarz N. Bless H. Strack F. Klumpp G. Rittenauer-Schatka H. Simons A. (1991). Ease of retrieval as information: Another look at the availability heuristic. Journal of Personality and Social Psychology, 61, 195–202.

[bibr50-01461672241233419] Selig J. P. Little T. D. (2012). Autoregressive and cross-lagged panel analysis for longitudinal data. In Laursen B. Little T. D. Card N. A. (Eds.), Handbook of developmental research methods (pp. 265–278). Guilford.

[bibr51-01461672241233419] Shrout P. E. Bolger N. (2002). Mediation in experimental and nonexperimental studies: New procedures and recommendations. Psychological Methods, 7, 422–445. 12530702

[bibr52-01461672241233419] Stryker S. (1980). Symbolic interactionism: A social structural version. Benjamin-Cummings.

[bibr53-01461672241233419] Swann W. B. Jr. (2012). Self-verification theory. In Van Lange P. Kruglanski A. Higgins E. T. (Eds.), Handbook of theories of social psychology (pp. 23–42). Sage.

[bibr54-01461672241233419] Swann W. B. Jr. De Le Ronde C. Hixon C. (1992). Embracing the bitter “truth”: Negative self-concepts and martial commitment. Psychological Science, 3, 118–121.

[bibr55-01461672241233419] Thai S. Lockwood P. (2015). Comparing you= comparing me: Social comparisons of the expanded self. Personality and Social Psychology Bulletin, 41, 989–1004. 26034204 10.1177/0146167215588094

[bibr56-01461672241233419] Thai S. Lockwood P. (2024). Social-judgment comparisons in daily life. Personality and Social Psychology Bulletin, 50, 38–57. 36052926 10.1177/01461672221115558

[bibr57-01461672241233419] Thai S. Lockwood P. Zhu R. Li Y. He J. C. (2019). The family ties that protect: Expanded-self-comparisons in parent-child relationships. Journal of Social and Personal Relationships, 36, 1041–1066.

[bibr58-01461672241233419] Van Breukelen G. J . (2006). ANCOVA versus change from baseline had more power in randomized studies and more bias in nonrandomized studies. Journal of Clinical Epidemiology, 59, 920–925. 16895814 10.1016/j.jclinepi.2006.02.007

[bibr59-01461672241233419] Vohs K. D. Aaker J. L. Catapano R. (2019). It’s not going to be that fun: Negative experiences can add meaning to life. Current Opinion in Psychology, 26, 11–14. 29704755 10.1016/j.copsyc.2018.04.014

[bibr60-01461672241233419] Walsh C. M. Neff L. A. (2018). We’re better when we blend: The benefits of couple identity fusion. Self and Identity, 17, 587–603.

[bibr61-01461672241233419] Weingarten E. Hutchinson J. (2018). Does ease mediate the ease-of-retrieval effect? A meta-analysis. Psychological Bulletin, 144, 227–283. 29389178 10.1037/bul0000122

