# Supplementary material for: On Creating Deeper Relationship Bonds: Felt Understanding Enhances Relationship Identification
Source: Pers Soc Psychol Bull. 2024 Mar 13;51(11):2248–65. doi: 10.1177/01461672241233419 (PMC12446700; doi:10.1177/01461672241233419)
Supplement: sj-docx-1-psp-10.1177_01461672241233419 – Supplemental material for On Creating Deeper Relationship Bonds: Felt Understanding Enhances Relationship Identification [file sj-docx-1-psp-10.1177_01461672241233419.docx]

**On Creating Deeper Relationship Bonds: Felt Understanding Enhances Relationship Identification**

**Supplemental Materials (SOM)**

| **Items** | | **Page** |
| --- | --- | --- |
| **Study 1** | |  |
|  | Original Study 1 | 2-6 |
|  | i. Scenarios | 2 |
|  | ii. Method and Results | 4 |
|  | Current Study 1 (Reported in Manuscript) | 7-12 |
|  | i. Models Without Covariates | 7 |
|  | ii. Mediation Analysis Results | 7 |
|  | iii. Gender and Relationship Effects (Tables S1-S2) | 10-12 |
| **Study 2** | |  |
|  | Attrition Analyses | 13 |
|  | Relationship Satisfaction Results | 14 |
|  | Over-Time Models Without Covariates | 15-16 |
|  | Inclusion-of-Other-in-Self vs. Identification Models Without Covariates | 16 |
|  | Gender Effects (Table S3) | 17 |
| **Study 3** | |  |
|  | Additional Details Regarding Participant Exclusions | 18 |
|  | Validation Studies for Ease-of-Retrieval Manipulation | 18-19 |
|  | Analyses with Multi-Item Measure of Understanding | 19-22 |
|  | Gender Effects (Tables S4 and S5) | 23-25 |
| **Study 4** | |  |
|  | Additional Details Regarding Participant Exclusions | 26 |
|  | Purpose and Mattering Results | 26 |
|  | Alternative Mediation Model Results | 26-27 |
|  | Gender Effects (Tables S6-S7) | 28-29 |
|  |  |  |
|  |  |  |
|  |  |  |
|  |  |  |
|  |  |  |
|  |  |  |
|  |  |  |
|  |  |  |
|  |  |  |
|  |  |  |
|  |  |  |
|  |  |  |
|  |  |  |
|  |  |  |
|  |  |  |
|  |  |  |
|  |  |  |

**Study 1: Person-Perception Paradigm**

**Original Study 1**

In our original submission, we used different scenarios for our person-perception paradigm. These scenarios are described below. We changed the scenarios because an anonymous reviewer noticed an asymmetry in the two scenarios that might be driving our results. More specifically, the overall perception of intimacy (feeling understood *and* cared for) was lower in the high caring/low understanding scenario compared to the high understanding/low caring scenario. We revised the scenarios to be more parallel and similar in the manuscript. We report the scenarios and results of the original Study 1 for interested readers below.

***Scenarios***

Participants were recruited to participate in a study on judgments and impressions of interpersonal relationships. To reduce participants’ suspicions about our hypotheses, they were told that they would be presented with short descriptions of two randomly selected types of social relationships. Participants were first presented with a short description of a coworker relationship. All participants read the same description:

“Alex and I are coworkers. We are both employed by a large marketing firm in Montreal. Alex has a degree in communications and I graduated with a degree in business administration. Alex and I sometimes travel to work together and we often pitch crazy ideas for ad campaigns to each other going to work. We share the same office space and enjoy emailing funny stories and jokes to each other. Alex and I can be very efficient when we work together; however, because we are both being considered for the same promotion we often prefer to work alone. Alex and I value the weekly lunches that we have together. If asked to describe my relationship with Alex in one word I would say it is professional.”

Then, participants were presented with one of the two versions of a vignette in which Jane describes her relationship with Mike. Approximately half of the participants were presented with a scenario in which Jane felt understood but not as cared for. The other half were presented with a scenario in which Jane felt cared for but not as understood. The high understanding/low caring relationship condition was described as follows:

“Mike and I have been in a dating relationship for the past three months. We are both young professionals living in the United States. I feel that no one knows me better than him. I feel that Mike has an intuitive understanding of who I am. He knows my deepest private goals, hopes, and fears better than anyone else does. I can say that he gets me. But at times, Mike can be a bit critical, questioning of me. He’s not the most conscientious person in the world so his support and caring is not totally reliable. I can completely count on him to understand me and my deepest concerns but there is not always follow through with support and caring.”

The high caring/low understanding relationship condition was described as follows:

“Mike and I have been in a dating relationship for the past three months. We are both young professionals living in the United States. I feel that Mike is very supportive and caring of me. He comforts me when I need it; he is just always there for me. I can say that he cares for me. But at times, I feel that he doesn’t know me. Mike doesn’t get my deepest private goals, hopes and fears. He just doesn’t get me at times. I can completely count on him to support me but he is a bit clueless and out of touch about some of the things that makes me “me”.

***Method***

**Participants.** One hundred and eighteen participants were recruited on Crowdflower to participate in a study on impressions of interpersonal relationships. Four participants were excluded because their citizenship made them ineligible (*n*=1) or they failed to complete the manipulation check or the dependent measure (*n*=3). This left 114 participants for data analysis (59 males, 55 females, *M*age=36.83, *SD*=13.36). In total, 69 were in a relationship (*M*_length_=12.25 years, *SD*=26.32), and 45 were single. Sensitivity analysis conducted with G*Power (Faul et al., 2007) revealed that, given our sample size, the minimum effect size that we could detect with a power of 80% and the alpha level at .05 was a Cohen’s *d* of .53 (equivalent to an *r* of .26) in a difference between two independent means.

**Procedure.** Participants were randomly assigned to read a scenario in which Jane felt understood but not as cared for (*n*=53) or a scenario in which Jane felt cared for but not as understood (*n*=61). Participants then indicated their perceptions of feeling understood and cared for (i.e., “How much do you think Jane *feels understood* by Mike?”; “How much do you think Jane feels *cared for* by Mike?”)—as defined by Reis and Shaver (1988), which served as our manipulation check. Next, participants indicated their perception of relationship identification using four items adapted from the Specific Relational-Interdependent Self-Construal (S-RISC) Scale (e.g., “To what extent do you think Jane feels Mike is an important part of who she is?”; Linardatos & Lydon, 2011; α=.84). We selected the items from the original scale that made the most sense for judging from a third-person perspective. All items were rated on a 7-point scale (1=*not at all* to 7=*very much*).

***Results***

**Manipulation Check.** A mixed 2x2 ANOVA (within: understanding and caring manipulation check items; between: high understanding vs. high caring) yielded a significant interaction between the items and the experimental condition; Wilks’=.48, *F*(1,112)=121.96, *p*<.001, *n*_p_^2^=.52). Ratings of understanding were higher in the high-understanding condition (*M*=5.43, *SD*=1.45) than in the high-caring condition (*M*=2.93, *SD*=1.40; *F*(1,112)=87.51, *p*<.001, *n*_p_^2^=.44). Ratings of caring were higher in the high-caring condition (*M*=5.77, *SD*=1.12) than in the high-understanding relationship (*M*=4.62, *SD*=1.11; *F*(1,112)=30.06, *p*<.001, *n*_p_^2^=.21).

**Relationship Identification.** A *t*-test yielded a significant main effect for condition, *t*(112)=3.55, *p*=.001, Cohen’s *d*=.67. Identification ratings were higher for the high-understanding relationship, *M*=4.82, *SD*=0.96, than for the high-caring relationship, *M*=4.12, *SD*=1.13.

We also explored the degree to which ratings of understanding and caring (i.e., the manipulation check measures) predicted relationship identification. Multiple regression revealed that higher understanding predicted higher relationship identification, *b*=0.29 [0.18, 0.39], *SE*=0.05, *t*(111)=5.67, *p*<.001, *r*=.47; however, caring was not reliably associated with relationship identification, *b*=0.13 [-0.05, 0.31], *SE*=0.08, *t*(111)=1.72, *p*=.09, *r*=.16.^[[1]](#footnote-1)^ This provides further support that understanding is uniquely associated with identification.

There were no gender or relationship status effects (*p*s>.07), these factors did not moderate any of the effects (*p*s>.13), and our effects remained significant when controlling for either status or gender (*ps*<.01).

**Satisfaction.** Because feeling cared for may also help individuals feel good about the relationship, we also examined differences in relationship satisfaction—that is, the extent to which people have positive feelings about the relationship. For exploratory purposes, participants also indicated their perceptions of relationship satisfaction using a single item (i.e., “How satisfied is Jane with the relationship?”; 1 = *Not at all* to 7 = *very much*).

We examined whether satisfaction levels differed between experimental conditions. Analysis revealed that satisfaction ratings were also higher for the high-understanding relationship (*M* = 4.62, *SD* = 1.04) than for the high-caring relationship (*M* = 3.98, *SD* = 1.15; *t*(112) = 3.10, *p* = .002, Cohen’s *d* = .58). In other words, Jane was perceived as being more identified with her relationship, but also more satisfied with her relationship when understanding was high than when caring was high.

We also conducted a multiple regression analysis to predict relationship satisfaction with the manipulation checks for understanding and caring entered as mean-centered continuous predictors. Both understanding, *b* = 0.36, BCa 95% CI [0.26, 0.46], *SE* = 0.05, *t*(111) = 7.47, *p* < .001, *r* = .58, and caring, *b* = 0.26, BCa 95% CI [0.13, 0.39], *SE* = 0.07, *t*(111) = 3.51, *p* < .001, *r* = .32, predicted relationship satisfaction.

This provides support that both understanding and caring may be important predictors of relationship satisfaction. Furthermore, this study provides evidence that understanding and caring may differentially impact satisfaction and identification in romantic relationships. Indeed, our main analyses revealed that only understanding uniquely predicted relationship identification.

**Current Study 1 (Reported in Manuscript)**

***Models Without Covariates***

We regressed relationship identification on understanding, relationship identification on caring, relationship identification on acceptance, and relationship identification on inclusion-of-other-in-self separately in four models. In the first model, greater understanding predicted greater identification, *b* = 0.26, *SE* = 0.03, *t*(259) = 7.71, *p* < .001. In the second model, caring did not predict identification, *b* = 0.02, *SE* = 0.05, *t*(259) = 0.44, *p* = .664. In the third model, greater acceptance predicted greater identification, *b* = 0.31, *SE* = 0.05, *t*(258) = 6.23, *p* < .001. In the fourth model, greater self-other overlap predicted greater identification, *b* = 0.60, *SE* = 0.05, *t*(257) = 11.95, *p* < .001.

***Mediation Analysis Results***

We first tested our mediation model (understanding 🡪 coherence 🡪 identification) using the PROCESS macro (model 4; Hayes, 2022) with 5000 bootstrap samples to estimate the indirect effects whereby experimental condition (high understanding/low caring vs. high caring/low understanding condition) influenced coherence which in turn predicted relationship identification. Participants perceived Jane to have a greater sense of coherence in the high understanding condition relative to the low understanding condition, *a*=0.72, 95% CI [0.39, 1.04], *SE*=0.16, *t*(259)=4.35, *p*<.001. Moreover, greater coherence predicted greater identification, *b*=0.37, 95% CI [0.27, 0.46], *SE*=0.05, *t*(258)=7.56, *p*<.001. This analysis also revealed that greater understanding affected relationship identification as a function of its relationship with coherence, *ab*=0.26, 95% CI [0.13, 0.41], *SE*=0.07. Although the total effect of experimental condition on relationship identification was significant, *c*=0.71, 95% CI [0.43, 0.99], *SE*=0.14, *t*(259)=5.04, *p*<.001, the direct effect of experimental condition on identification was reduced when the indirect effect through coherence was taken into account, *c’*=0.45, 95% CI [0.19, 0.71], *SE*=0.13, *t*(258)=3.40, *p*=.001.

Next, we conducted our mediation analysis using ratings of understanding while controlling for acceptance and caring because caring also varied between the two experimental conditions. Although acceptance did not vary between the two conditions, we controlled for it to demonstrate the unique effect of understanding, which would be consistent with our hypothesis. In this model, greater understanding still predicted greater coherence after controlling for caring and acceptance, *a*=0.28 95% CI [0.19, 0.37], *SE*=0.05, *t*(256)=6.18, *p*<.001. Moreover, greater coherence predicted greater identification after controlling for caring and acceptance, *b*=0.27 95% CI [0.17, 0.37], *SE*=0.05, *t*(255)=5.36, *p*<.001. This analysis also revealed that greater understanding affected relationship identification as a function of its relationship with coherence, *ab*=0.08, 95% CI [0.04, 0.12], *SE*=0.02, after controlling for caring and acceptance. Although the total effect of understanding on relationship identification was significant after controlling for caring and acceptance, *c*=0.21, 95% CI [0.13, 0.28], *SE*=0.04, *t*(256)=5.40, *p*<.001, the direct effect of understanding on identification was reduced when the indirect effect through coherence was taken into account, *c’*=0.13, 95% CI [0.05, 0.21], *SE*=0.04, *t*(255)=3.38, *p*=.001.

In our final model, we controlled for caring, acceptance, and inclusion-of-other-in-self. In this model, greater understanding still predicted greater coherence after controlling for caring, acceptance, and inclusion-of-other-in-self, *a*=0.25 95% CI [0.15, 0.34], *SE*=0.05, *t*(253)=5.20, *p*<.001. Moreover, greater coherence predicted greater identification after controlling for caring, acceptance, and inclusion-of-other-in-self, *b*=0.21 95% CI [0.12, 0.30], *SE*=0.05, *t*(252)=4.63, *p*<.001. This analysis also revealed that greater understanding affected relationship identification as a function of its relationship with coherence, *ab*=0.05, 95% CI [0.02, 0.09], *SE*=0.02, after controlling for caring, acceptance, and inclusion-of-other-in-self. Although the total effect of understanding on relationship identification was significant after controlling for caring, acceptance, and inclusion-of-other-in-self, *c*=0.11, 95% CI [0.04, 0.18], *SE*=0.04, *t*(253)=3.05, *p*=.003, the direct effect of understanding on identification was no longer significant when the indirect effect through coherence was taken into account, *c’*=0.06, 95% CI [-0.01, 0.13], *SE*=0.04, *t*(252)=1.58, *p*=.116.

**Table S1**

*Gender and Relationship Effects for Study 1 (Regression Models)*

| Effect | | *b* | *SE* | 95% CI | | *p* | *r* |
| --- | --- | --- | --- | --- | --- | --- | --- |
|  |  |  |  | *LL* | *UL* |  |  |
| Model 1 (Identification as DV) | |  |  |  |  |  |  |
|  | Understanding (Continuous predictor) | 0.28 | 0.03 | 0.21 | 0.35 | < .001 | .46 |
|  | Caring (Continuous predictor) | 0.10 | 0.04 | 0.01 | 0.19 | .027 | .14 |
|  | Gender | -0.05 | 0.07 | -0.18 | 0.08 | .466 | .05 |
|  | Understanding X Gender | 0.07 | 0.03 | 0.00 | 0.14 | .053 | .12 |
|  | Caring X Gender | 0.02 | 0.04 | -0.07 | 0.10 | .679 | .03 |
|  |  |  |  |  |  |  |  |
| Model 2 (Identification as DV) | |  |  |  |  |  |  |
|  | Understanding (Continuous predictor) | 0.28 | 0.04 | 0.20 | 0.37 | < .001 | 0.41 |
|  | Caring (Continuous predictor) | 0.11 | 0.05 | 0.01 | 0.22 | .032 | 0.13 |
|  | Relationship Status | -0.03 | 0.07 | -0.17 | 0.11 | .682 | 0.03 |
|  | Understanding X Relationship Status | -0.01 | 0.04 | -0.10 | 0.07 | .779 | 0.02 |
|  | Caring X Relationship Status | -0.02 | 0.05 | -0.13 | 0.08 | .665 | 0.03 |
|  |  |  |  |  |  |  |  |
| Model 3 (Acceptance as DV) | |  |  |  |  |  |  |
|  | Scenario Condition  (High Caring vs. High Understanding) | 0.02 | 0.08 | -0.15 | 0.18 | .811 | .02 |
|  | Gender | 0.26 | 0.08 | 0.09 | 0.42 | .002 | .19 |
|  | Scenario X Gender | 0.04 | 0.08 | -0.13 | 0.20 | .678 | .03 |
|  |  |  |  |  |  |  |  |
| Model 4 (Acceptance as DV) | |  |  |  |  |  |  |
|  | Scenario Condition  (High Caring vs. High Understanding) | -0.01 | 0.09 | -0.18 | 0.16 | .906 | .01 |
|  | Relationship Status | 0.03 | 0.09 | -0.14 | 0.20 | .707 | .02 |
|  | Scenario X Relationship Status | 0.10 | 0.09 | -0.07 | 0.27 | .286 | .07 |
|  |  |  |  |  |  |  |  |
| Model 5 (Satisfaction as DV) | |  |  |  |  |  |  |
|  | Scenario Condition  (High Caring vs. High Understanding) | 0.00 | 0.07 | -0.13 | 0.15 | .951 | .00 |
|  | Gender | 0.03 | 0.07 | -0.11 | 0.17 | .629 | .03 |
|  | Scenario X Gender | -0.08 | 0.07 | -0.23 | 0.06 | .244 | .07 |
|  |  |  |  |  |  |  |  |
| Model 6 (Satisfaction as DV) | |  |  |  |  |  |  |
|  | Scenario Condition  (High Caring vs. High Understanding) | -0.04 | 0.07 | -0.18 | 0.10 | .597 | .03 |
|  | Relationship Status | 0.12 | 0.07 | -0.02 | 0.26 | .095 | .10 |
|  | Scenario X Relationship Status | 0.16 | 0.07 | 0.02 | 0.30 | .026 | .14 |
|  | Simple Effects |  |  |  |  |  |  |
|  | Relationship Status Effect   in Understanding Condition | 0.29 | 0.10 | 0.09 | 0.48 | .006 | .17 |
|  | Relationship Status Effect  in Caring Condition | -0.04 | 0.10 | -0.24 | 0.17 | .693 | .02 |

*Note.* Gender was effects-coded as -1 women, +1 men. Relationship status was effects coded as
-1 single and +1 in a relationship. *LL* = lower limit. *UL* = upper limit.

**Table S2**

*Gender and Relationship Status Effects for Study 1 (ANOVA models)*

| Effect | | *df* | *F* | *p* | *n*_p_^2^ |
| --- | --- | --- | --- | --- | --- |
| Manipulation Check | |  |  |  |  |
|  | Scenario Condition (High Caring vs. High Understanding) | 1, 256 | 19.33 | < .001 | .070 |
|  | Gender (Men vs. Women) | 1, 256 | 3.87 | .050 | .015 |
|  | Responsiveness Rating (Understanding vs. Caring) | 1, 256 | 41.21 | < .001 | .139 |
|  | Scenario X Gender | 1, 256 | 0.40 | .527 | .002 |
|  | Scenario X Responsiveness | 1, 256 | 348.44 | <.001 | .576 |
|  | Gender X Responsiveness | 1, 256 | 2.51 | .114 | .010 |
|  | Scenario X Gender X Responsiveness | 1, 256 | 0.22 | .639 | < .001 |
|  |  |  |  |  |  |
| Manipulation Check | |  |  |  |  |
|  | Scenario Condition (High Caring vs. High Understanding) | 1, 257 | 15.00 | <.001 | .055 |
|  | Relationship Status (Single vs. In Relationship) | 1, 257 | 0.49 | .483 | .002 |
|  | Responsiveness Rating (Understanding vs. Caring) | 1, 257 | 38.26 | <.001 | .130 |
|  | Scenario X Status | 1, 257 | 1.35 | .247 | .005 |
|  | Scenario X Responsiveness | 1, 257 | 343.73 | <.001 | .572 |
|  | Status X Responsiveness | 1, 257 | 0.08 | .776 | <.001 |
|  | Scenario X Status X Responsiveness | 1, 257 | 3.06 | .081 | .012 |
|  |  |  |  |  |  |

**Study 2: Two-Wave Longitudinal Study**

**Attrition Analyses**

First, we tested for differences between participants who returned at Time 2 but reported a change in relationship status. That is, we tested whether participants who reported breaking up at Time 2 differed from those who reported still being in the same relationship at Time 2. Participants who broke up (*n*=27) did not differ on understanding, *t*(51.51)=1.38, *p*=.174, acceptance, *t*(53.28)=-0.76, *p*=.453, or caring, *t*(49.78)=1.27, *p*=.211, from those who stayed with the same romantic partner at Time 2. However, consistent with Linardatos and Lydon (2011), participants who broke up had significantly lower levels of identification at Time 1 (*M* = 4.51, *SE* = 0.22) than those who stayed together (*M* = 5.16, *SE* = 0.10), *t*(37.27)=2.63, *p*=.012.

Next, we tested whether those who did *not* complete the Time 2 survey differed from those who did complete the survey and were still in the same relationship at Time 2. Participants who did not return at Time 2 (*n* = 52) did not differ on identification, *t*(108.09) = 1.63, *p* = .107, acceptance, *t*(85.69) = 1.28, *p* = .203, or caring, *t*(93.03) = 1.25, *p* = .216. These participants, however, reported lower understanding (*M* = 5.12, *SE* = 0.21) at Time 1 than participants who completed Time 2 and were still in the same relationship (*M* = 5.68, *SE* = 0.14), *t*(97.20) = 2.26, *p* = .026. The impact of these difference on our overall results should be minimal because the full range of understanding skills was still represented in the sample of participants who were still in the same relationship and completed the survey at Time 2 (i.e., understanding range = 1 to 7). Moreover, we used the full sample, which included these participants, when estimating our cross-lagged panel model (CLPM) to determine the temporal precedence of understanding.

**Relationship Satisfaction Results**

In Study 2, we also tested for discriminant validity by examining whether feeling understood predicted relationship satisfaction, controlling for feeling cared for and accepted. At Time 1 and Time 2, participants completed a relationship general form of the Quality of Marriage Index (Norton, 1983). The scale consists of six items including “My relationship with my partner makes me happy” and “We have a good relationship” (α = .95 at Time 1 and α = .96 at Time 2; 1 = *very strongly disagree* to 7 = *very strongly agree*)*.* The last item of this scale asked participants to indicate what degree of happiness best described their relationship when all things were considered (1 = *unhappy* to 10 = *perfectly happy*, the middle point being *happy*). Items were standardized and averaged to create a composite score of relationship satisfaction. We found that none of the perceived partner responsiveness components uniquely predicted satisfaction at Time 2, *z*s < 1.06, *p*s > .291, controlling for satisfaction at Time 1.

These findings extend the results of Study 1 by showing that feeling understood by one’s partner predicts relationship identification over time, despite the overall high stability of identification. Moreover, because perceived understanding predicts relationship identification after controlling for feeling cared for and accepted, this provides further evidence that there is something unique about perceived understanding that promotes relationship identification. The present findings also provide discriminant validity: Feeling understood at Time 1 was associated with relationship identification at Time 2 after controlling for baseline identification; however, feeling understood at Time 1 was not reliably associated with relationship satisfaction at Time 2 after controlling for baseline satisfaction. Moreover, none of the perceived partner responsiveness components uniquely contributed to the prediction of relationship satisfaction.

**Over-Time Models Without Covariates**

We repeated the temporal precedence analyses for acceptance and identification and then caring and identification to explore the effects of acceptance on identification and caring on identification separately (i.e., without controlling for other components of perceived responsiveness).

***Acceptance and Identification***

Model 1 included only the autoregressive effects (paths *a* and *b*). This model fits the data, χ^2^(2)=4.84, *p*=.089, TLI=0.96, RMSEA=0.09 CI_90%_ [0.00, 0.19], SRMR=0.05. In Model 2, we added the cross-lagged path between Time 1 acceptance and Time 2 identification (path *c*). This model fits the data, χ^2^(1)=2.41, *p*=.120, TLI=0.96, RMSEA=0.08 CI_90%_ [0.00, 0.23], SRMR=0.03, and did not fit the data better than Model 1, ∆χ^2^(1)=2.43, *p*=.119. However, acceptance at Time 1 did not predict identification at Time 2 after controlling for identification at Time 1, *c* = 0.10, *SE* = 0.06, *z* = 1.57, *p* = .118. In Model 3, we included the two autoregressive paths (paths *a* and *b*) and the cross-lagged path between Time 1 identification and Time 2 acceptance (path *d*). This model had a relatively good fit, χ^2^(1)=2.15, *p*=.143, TLI=0.97, RMSEA=0.08 CI_90%_ [0.00, 0.22], SRMR=0.03. However, the cross-lagged path from Time 1 identification to Time 2 acceptance while controlling for Time 1 acceptance was not significant, *d*=0.11, *SE*=0.07, *p*=.099. Model 3 did not fit the data better than Model 1, ∆χ^2^(1)=2.70, *p*=.101. Thus, acceptance did not precede identification.

***Caring and Identification***

Model 1 included only the autoregressive effects (paths *a* and *b*). This model did not fit the data well, χ^2^(2)=11.83, *p*=.003, TLI=0.89, RMSEA=0.16 CI_90%_ [0.08, 0.25], SRMR=0.07. In Model 2, we added the cross-lagged path between Time 1 caring and Time 2 identification (path *c*). This model did not fit the data, χ^2^(1)=8.55, *p*=.003, TLI=0.83, RMSEA=0.20 CI_90%_ [0.09, 0.33], SRMR=0.05, and did not fit the data better than Model 1, ∆χ^2^(1)=3.28, *p*=.070. In addition, caring at Time 1 did not predict identification at Time 2 while controlling for identification at Time 1, *c* = 0.12, *SE* = 0.07, *z* = 1.82, *p* = .069. In contrast, Model 3, which included the two autoregressive paths (paths *a* and *b*) and the cross-lagged path between Time 1 identification and Time 2 caring (path *d*), had a relatively good fit, χ^2^(1)=2.52, *p*=.113, TLI=0.97, RMSEA=0.09 CI_90%_ [0.00, 0.23], SRMR=0.03. Moreover, this cross-lagged path was significant, *d*=0.17, *SE*=0.05, *p*=.002, and Model 3 did fit the data better than Model 1, ∆χ^2^(1)=9.31, *p*=.002. Thus, these results suggest greater identification predicts greater caring. Furthermore, there is limited evidence suggesting that caring precedes identification.

**Inclusion-of-Other-in-Self vs. Identification Models Without Covariates**

For each timepoint, we tested four different models regressing identification on each predictor separately (understanding, caring, acceptance, inclusion-of-other-in-self). At Time 1, greater understanding predicted greater identification, *b* = 0.38, *SE* = 0.06, *t*(115) = 6.42, *p* < .001. At Time 1, greater caring also predicted greater identification, *b* = 0.36, *SE* = 0.09, *t*(114) = 4.25, *p* < .001. At Time 1, greater acceptance predicted greater identification, *b* = 0.31, *SE* = 0.09, *t*(115) = 3.57, *p* < .001. At Time 1, greater self-other overlap predicted greater identification, *b* = 0.44, *SE* = 0.06, *t*(115) = 7.16, *p* < .001. We also replicated these associations at Time 2. At Time 2, greater understanding predicted greater identification, *b* = 0.33, *SE* = 0.07, *t*(114) = 4.89, *p* < .001. At Time 2, greater caring also predicted greater identification, *b* = 0.47, *SE* = 0.09, *t*(115) = 5.34, *p* < .001. At Time 2, greater acceptance predicted greater identification, *b* = 0.33, *SE* = 0.09, *t*(115) = 3.63, *p* < .001. At Time 2, greater self-other overlap predicted greater identification, *b* = 0.31, *SE* = 0.08, *t*(115) = 4.18, *p* < .001.

**Table S3**

*Gender Effects for Study 2*

|  |  |  |  | 95% CI | |  |  |
| --- | --- | --- | --- | --- | --- | --- | --- |
|  | Effects | *b* | *SE* | *LL* | *UL* | *p* | *r* |
| Time 1 Model  (Time 1 Identification as Outcome) | |  |  |  |  |  |  |
|  | Time 1 IOS | 0.17 | 0.08 | -0.03 | 0.35 | .043 | .20 |
|  | Time 1 Understanding | 0.20 | 0.12 | -0.08 | 0.44 | .098 | .16 |
|  | Time 1 Caring | 0.12 | 0.15 | -0.15 | 0.54 | .430 | .08 |
|  | Time 1 Acceptance | 0.01 | 0.15 | -0.36 | 0.36 | .930 | .01 |
|  | Gender | 0.17 | 0.09 | -0.02 | 0.33 | .079 | .17 |
|  | Time 1 IOS X Gender | -0.21 | 0.08 | -0.42 | -0.03 | .011 | .24 |
|  | Simple Effect of IOS for Men | -0.04 | 0.14 | -0.40 | 0.25 | .752 | .03 |
|  | Simple Effect of IOS for Women | 0.39 | 0.09 | 0.18 | 0.59 | <.001 | .37 |
|  | Time 1 Understanding X Gender | -0.14 | 0.12 | -0.39 | 0.11 | .242 | .11 |
|  | Time 1 Caring X Gender | 0.13 | 0.15 | -0.15 | 0.52 | .387 | .08 |
|  | Time 1 Acceptance X Gender | 0.24 | 0.15 | -0.14 | 0.58 | .109 | .15 |
|  |  |  |  |  |  |  |  |
| Time 2 Model  (Time 2 Identification as Outcome) | |  |  |  |  |  |  |
|  | Time 2 IOS | 0.25 | 0.08 | 0.09 | 0.44 | .002 | .29 |
|  | Time 2 Understanding | 0.26 | 0.11 | 0.05 | 0.56 | .023 | .22 |
|  | Time 2 Caring | 0.21 | 0.18 | -0.19 | 0.52 | .224 | .12 |
|  | Time 2 Acceptance | -0.07 | 0.17 | -0.47 | 0.21 | .663 | .04 |
|  | Gender | 0.20 | 0.10 | -0.03 | 0.37 | .043 | .20 |
|  | Time 2 IOS X Gender | -0.12 | 0.08 | -0.26 | 0.07 | .127 | .15 |
|  | Time 2 Understanding X Gender | -0.08 | 0.11 | -0.29 | 0.22 | .498 | .07 |
|  | Time 2 Caring X Gender | 0.01 | 0.18 | -0.40 | 0.33 | .941 | .01 |
|  | Time 2 Acceptance X Gender | 0.12 | 0.17 | -0.29 | 0.38 | .497 | .07 |

*Note*. Significant gender interactions should be interpreted with caution due to the gender distribution in the study (31 men, 86 women). Gender was effects coded as +1 men and -1 women. *LL* = lower limit. *UL* = upper limit.

**Study 3: Ease-of-Retrieval Manipulation**

**Additional Details Regarding Participant Exclusions**

We examined participants’ open-ended responses following the manipulation to ensure that they understood and followed the instructions. Four participants in the understanding condition reported instances revealing a lack of understanding, two participants in the easy condition reported fewer than three instances, and two participants wrote single word responses. Participants who do not follow instructions increase statistical noise, and thus decrease statistical power (Goodman et al., 2013). For this reason, these participants along with those who failed the directed question were excluded from analyses. We also used a single directed question in the pretest and posttest questionnaire (“This is a control question. Leave this question blank.”; Maniaci & Rogge, 2014) due to the short length of the survey. Participant who answered this question were excluded.

**Validation Studies for Ease-of-Retrieval Manipulation**

We conducted two separate manipulation check studies to determine how difficult it was for individuals to recall instances in which they felt understood. For these studies, we recruited people in a relationship and singles from MTurk. Participants in a relationship answered questions about their romantic partner, and singles answered questions about a close friend. We first examined how many instances of partner (or friend) understanding individuals could freely generate. Pretesting conducted among romantically-involved individuals (*N* = 46) indicated that 70% of the participants could freely generate between one and three instances; the modal response was three instances. No one reported more than nine instances of partner understanding. Among participants who were singles (*N* = 38), 95% of the participants could freely generate between one and three instances; the modal response was two instances. No one reported more than four.

We conducted a second manipulation check study to examine how difficult it would be to generate three vs. nine instances. Romantically involved participants (*N* = 46) tended to have more difficulty generating nine instances (*M* = 5.90; *SD* = 2.66) than three instances (*M* = 4.28; *SD* = 2.87; 1 = *not at all difficult* to 10 = *very difficult*; *t*(44) = -1.98, *p* = .054). Furthermore, singles (*N* = 21) found it more difficult to generate nine instances (*M* = 8.00; *SD* = 2.00) than three (*M* = 5.25; *SD* = 2.92; *t*(19) = -2.57, *p* = .019). Thus, these findings suggest that recalling three instances may be relatively easy, whereas recalling nine instances may be relatively difficulty. However, these findings suggest that the ease manipulation may be more powerful for friends than partners.

**Analyses with Multi-Item Measure of Understanding**

In Study 3, we also included a multi-item measure of understanding (Campbell et al., 2006) in the pretest questionnaire that included items like “My partner/friend knows me better than anyone else,” and “My partner/friend and I can accurately predict each other’s behaviour in different situations” (see page 9 of Materials Supplement for full scale). These items were rated on a scale ranging from 1 (*Disagree strongly)* to 9 (*Agree strongly*). In a pilot study with 52 participants, the single item of understanding reported in the manuscript was highly correlated with this multi-item scale (*r* = .74, *p* = .001). They were also highly correlated in Study 3 (*r* = .76, *p* < .001). When we used the multi-item scale in our Study 3 analyses, we obtained the same pattern of results. These results are reported below.

***Direct Effects***

To determine group differences in understanding, we regressed Time 2 understanding using the single-item measure on recall condition, relationship type, and their interaction while controlling for baseline understanding using the multi-item scale. Participants felt more understood in the easy than difficult condition, *b* = 0.15 [0.01, 0.30], *SE* = 0.07, *t*(352) = 2.17, *p* = .030, *r* = .12. Unexpectedly, participants also felt more understood in the friend than partner condition, *b* = -0.64 [-0.79, -0.48], *SE* = 0.07, *t*(352) = -8.57, *p* < .001, *r* = .42. These effects, however, were qualified by a significant recall-by-relationship interaction, *b* = -0.17 [-0.31, -0.02], *SE* = 0.07, *t*(352) = -2.37, *p* = .018, *r* = .13. In the friend condition, participants felt more understood in the easy (*M*=8.74, *SE*=0.18) than difficult condition (*M*=8.10, *SE*=0.17), *b* = 0.32 [0.06, 0.58], *SE* = 0.12, *t*(352) = 2.66, *p* = .008, *r* = .14. In contrast, recall conditions did not differ in the partner condition, *b* = -0.01 [-0.15, 0.12], *SE* = 0.07, *t*(352) = -0.19, *p* = .848, *r* = .01.

Because we lacked a true control condition, we then examined how understanding changed from pretest to posttest to determine the direction of the manipulation using a 2x2x2 mixed ANOVA (within: pretest-posttest; between: recall condition, relationship type) and report results regarding the change from pretest to posttest only. There was a significant recall condition-by-time interaction, *F*(1,353)=4.53, *p*=.034, *n*_p_^2^=.01. In both the easy condition, *t*(353) = 6.31, *p* < .001, and difficult condition, *t*(353) = -3.67, *p* < .001, understanding increased from pretest to posttest; however, this increase was larger in the easy condition. There was also a significant relationship type-by-time interaction, *F*(1, 353)=107.45, *p*<.001, *n*_p_^2^=.23. In the friend condition, understanding increased from pretest (*M* = 6.62, *SE* = 0.14) to posttest (*M* = 7.91, *SE* = 0.16), *t*(353) = 10.25, *p* < .001; however, in the partner condition, understanding decreased from pretest (*M* = 7.58, *SE* = 0.08) to posttest (*M* = 7.34, *SE* = 0.10), *t*(353) = -3.11, *p* = .002. Because the pretest and posttest measures of understanding differed, it is unclear how we should interpret these changes in understanding.

***Mediational Analysis***

Several researchers have highlighted that a manipulation can influence an outcome indirectly even when the direct effect is not significant (Hayes & Rockwood, 2017; Shrout & Bolger, 2002). Thus, the recall condition may have affected the psychological mechanisms by which it was expected to impact identification (i.e., through the indirect effect of recall on subjective ease and understanding). To examine this possibility, we estimated this indirect effect simultaneously for friends and partners. Using a multi-group path analysis and the FIML estimation procedure, we tested whether the indirect effect varied between relationship types (Kline, 2015; Ryu & Cheong, 2017). Consistent with our previous analyses, we included pretest understanding and identification as covariates. A Likelihood Ratio Test revealed that the indirect effect of the recall condition, through subjective ease and felt understanding, on identification did not differ between relationship types, *χ*^2^(3)=3.19, *p*=.363. Therefore, the indirect paths were estimated using the pooled data. The paths from pretest understanding and pretest identification to subjective ease and the path from recall condition to posttest understanding varied as a function of relationship type and were thus freely estimated. The intercepts and residuals variances of subjective ease, posttest understanding, and posttest identification also varied and were also freely estimated.

Participants who generated fewer instances of understanding reported greater subjective ease, *a_1_* = 2.02 [1.45, 2.57], *SE* = 0.29, *z* = 7.01, *p* < .001, and participants who found it easier to generate examples felt more understood, *d* = 0.10 [0.05, 0.15], *SE* = 0.03, *z* = 3.83, *p* <.001. Participants who felt more understood post-manipulation also reported greater identification, controlling for pretest understanding and identification, *b_2_* = 0.10 [0.04, 0.17], *SE* = 0.03, *z* = 3.09, *p* = .002. The indirect effect of recall condition, through subjective ease and understanding, on identification, was significant, *a_1_db_2_ =* 0.02 [0.01, 0.05]. Although the direct effect of recall condition on understanding (*a*_2_) differed between relationship type, *χ*^2^(1)=4.04, *p*=.044, neither the path for friends nor partner reached significance, *z*s<1.55, *p*s>.12.

**Table S4**

*Gender Effects for Study 3 (Regression Models)*

| Effect | | *b* | *SE* | 95% CI | | *p* | *r* |
| --- | --- | --- | --- | --- | --- | --- | --- |
|  |  |  |  | *LL* | *UL* |  |  |
| Subjective Ease as DV | |  |  |  |  |  |  |
|  | Recall Condition (Easy vs. Hard) | 1.16 | 0.17 | 0.84 | 1.48 | <.001 | .35 |
|  | Relationship Type (Friend vs. Partner) | 0.51 | 0.17 | 0.19 | 0.83 | .002 | .16 |
|  | Gender (Men vs. Women) | -0.11 | 0.17 | -0.42 | 0.21 | .529 | .03 |
|  | Recall X Relationship | -0.24 | 0.17 | -0.54 | 0.07 | .158 | .08 |
|  | Recall X Gender | 0.43 | 0.17 | 0.12 | 0.76 | .010 | .14 |
|  | Simple effect of recall condition for   men | 1.59 | 0.25 | 1.14 | 2.04 | <.001 | .33 |
|  | Simple effect of recall condition for   women | 0.73 | 0.22 | 0.28 | 1.17 | .001 | .17 |
|  | Relationship X Gender | 0.12 | 0.17 | -0.20 | 0.44 | .461 | .04 |
|  | Recall X Relationship X Gender | -0.07 | 0.17 | -0.39 | 0.25 | .682 | .02 |
|  |  |  |  |  |  |  |  |
| Understanding as DV | |  |  |  |  |  |  |
|  | Baseline Understanding | 0.70 | 0.04 | 0.60 | 0.79 | <.001 | .70 |
|  | Recall Condition | 0.17 | 0.07 | 0.03 | 0.31 | .011 | .14 |
|  | Relationship Type | -0.35 | 0.07 | -0.49 | -0.19 | <.001 | .28 |
|  | Gender | 0.12 | 0.07 | -0.03 | 0.26 | .077 | .10 |
|  | Recall X Relationship Type | -0.15 | 0.07 | -0.29 | 0.00 | .027 | .12 |
|  | Recall X Gender | 0.16 | 0.07 | 0.02 | 0.30 | .016 | .14 |
|  | Simple effect of recall condition for   men | 0.33 | 0.10 | 0.12 | 0.55 | .001 | .19 |
|  | Simple effect of recall condition for   women | 0.01 | 0.09 | -0.19 | 0.20 | .922 | .01 |
|  | Relationship X Gender | -0.06 | 0.07 | -0.20 | 0.09 | .398 | .05 |
|  | Ease X Relationship X Gender | -0.08 | 0.07 | -0.22 | 0.06 | .217 | .07 |
|  |  |  |  |  |  |  |  |
| Identification as DV | |  |  |  |  |  |  |
|  | Baseline Identification | 0.67 | 0.03 | 0.59 | 0.76 | <.001 | .75 |
|  | Recall Condition (Easy vs. Hard) | 0.05 | 0.04 | -0.04 | 0.13 | .239 | .06 |
|  | Relationship Type (Friend vs. Partner) | 0.12 | 0.04 | 0.03 | 0.20 | .004 | .15 |
|  | Gender (Men vs. Women) | 0.01 | 0.04 | -0.08 | 0.09 | .876 | .01 |
|  | Recall X Relationship | -0.11 | 0.04 | -0.20 | -0.02 | .006 | .15 |
|  | Recall X Gender | 0.03 | 0.04 | -0.06 | 0.11 | .423 | .04 |
|  | Relationship X Gender | 0.04 | 0.04 | -0.04 | 0.12 | .298 | .06 |
|  | Ease X Relationship X Gender | -0.06 | 0.04 | -0.15 | 0.02 | .125 | .08 |

*Note.* Gender was effects-coded as -1 women, +1 men. *LL* = lower limit. *UL* = upper limit.

**Table S5**

*Gender Effects for Study 3 (ANOVA Models)*

|  | Effect | *df* | *F* | *p* | *n*_p_^2^ |
| --- | --- | --- | --- | --- | --- |
| Change from Pretest to Posttest Understanding | |  |  |  |  |
|  | Recall Condition (Easy vs. Hard) | 1, 314 | 1.45 | .230 | .005 |
|  | Relationship Type (Friend vs. Partner) | 1, 314 | 0.00 | .956 | < .001 |
|  | Gender (Men vs. Women) | 1, 314 | 0.17 | .684 | < .001 |
|  | Time (Pretest vs. Posttest) | 1, 314 | 78.11 | <.001 | .20 |
|  | Recall X Relationship | 1, 314 | 3.67 | .056 | .012 |
|  | Recall X Gender | 1, 314 | 4.36 | .038 | .014 |
|  | Relationship X Gender | 1, 314 | 7.83 | .005 | .024 |
|  | Recall X Relationship X Gender | 1, 314 | 1.28 | .259 | .004 |
|  | Recall X Time | 1, 314 | 5.07 | .025 | .016 |
|  | Relationship X Time | 1, 314 | 31.65 | < .001 | .092 |
|  | Gender X Time | 1, 314 | 3.08 | .080 | .010 |
|  | Recall X Relationship X Time | 1, 314 | 2.55 | .112 | .008 |
|  | Recall X Gender X Time | 1, 314 | 2.99 | .085 | .009 |
|  | Relationship X Gender X Time | 1, 314 | 4.56 | .034 | .014 |
|  | Recall X Relationship X Gender X Time | 1, 314 | 0.74 | .390 | .002 |

**Simple Effects Analyses**

***Recall Condition by Gender Interaction***

For women, there was no significant difference between recall conditions, *t*(314) = 0.66, *p* = .516. For men, there was a significant difference between recall conditions, *t*(314) = - 2.22, *p* = .027, such that men in the easy condition felt more understood (*M* = 7.64, *SE* = 0.18) than men in the difficult condition (*M* = 7.07, *SE* = 0.18). There was also no gender difference in the easy condition, *t*(314) = 1.74, *p* = .084, or in the difficult condition, *t*(314) = -1.21, *p* = .228.

***Relationship by Gender Interaction***

For women, there was a significant difference between the friend and partner conditions for understanding, *t*(314) = 2.12, *p* = .034. Women felt more understood by their friend (*M* = 7.53, *SE* = 0.20) than their partner (*M* = 7.04, *SE* = 0.12). For men, there was no significant difference between the friend and partner conditions for understanding, *t*(314) = -1.85, *p* = .065. For the friend condition, men and women did not differ in how understood they felt, *t*(314) = -1.42, *p* = .156. For the partner condition, men and women did differ significantly, *t*(314) = 2.95, *p* = .004. Men felt more understood by their partner (*M* = 7.59, *SE* = 0.15) than women (*M* = 7.04, *SE* = 0.12). These results, however, should be interpreted with caution because it is qualified by a significant relationship-by-gender-by-time interaction.

***Relationship by Gender by Time Interaction***

To decompose this interaction, we used multilevel models. For both men, *b* = -0.58, *SE* = 0.11, *t*(321.29) = -5.38, *p* < .001, and women, *b* = -0.26, *SE* = 0.10, *t*(321.71) = -2.67, *p* = .008, there was a significant time-by-relationship type interaction. For men in the friend condition, understanding increased from pretest to posttest, *b* = 1.36, *SE* = 0.17, *t*(322.19) = 7.79, *p* < .001. For men in the partner condition, there was no change in pretest to posttest understanding, *b* = 0.21, *SE* = 0.13, *t*(319.54) = 1.64, *p* = .101. In contrast, women reported understanding increasing from pretest to posttest in both the friend, *b* = 0.78, *SE* = 0.17, *t*(320.32) = 4.63, *p* < .001, and partner, *b* = 0.26, *SE* = 0.10, *t*(326.11) = 2.75, *p* = .006, conditions.

**Study 4: Visualization Manipulation**

**Additional Details Regarding Participant Exclusions**

We examined participants’ open-ended responses following the manipulation. Eight participants could not think of an event. Seventeen participants in the low understanding condition said their close other would understand them. Thirty participants in the high understanding condition said their close other could not understand them.

**Purpose and Mattering Results**

We also examined whether changes in understanding caused changes in the other facets of meaning-in-life, purpose and mattering, by regressing each of these facets on visualization condition, relationship type, their interaction, and the pretest measure of the facet. There were no significant effects or interactions of visualization condition and relationship type on purpose, *t*s<1.28, *p*s>.20. There were also no significant effects or interactions of visualization condition and relationship type on mattering, *t*s<1.37, *p*s>.17.

**Alternative Mediation Model Results**

We argued that when individuals feel understood, it may change how they see themselves. It is also possible, however, that developing a strong sense of relationship identification increases coherence because individuals use their identity to make sense of the world (Thoits, 1983; McGregor & Little, 1998). We tested this alternative model and found that the indirect effect of condition, through felt understanding and relationship identification, on comprehension was also plausible, *Indirect* *Path* *a_1_db_2_*=0.03, 95% bootstrap CI [0.01, 0.05]. In this alternative model, pretest understanding and identification were included as covariates. Some paths for these covariates varied between relationship type and were thus freely estimated. These results suggest that the visualization manipulation may have increased coherence indirectly by affecting felt understanding and relationship identification, an effect that did not differ across relationship type, *χ*^2^(3)=2.53, *p*=.469. We note, however, that this reverse mediation approach has been shown to be uninformative in distinguishing between two alternative causal models (Lemmer & Gollwitzer, 2017).

**Table S6**

*Gender Effects for Study 4 (Regression Models)*

| Effects | | *b* | *SE* | 95% CI | | *p* | *r* |
| --- | --- | --- | --- | --- | --- | --- | --- |
|  |  |  |  | *LL* | *UL* |  |  |
| Understanding as Outcome | |  |  |  |  |  |  |
|  | Baseline Understanding | 0.60 | 0.04 | 0.49 | 0.71 | <.001 | .64 |
|  | Visualization Condition | 0.43 | 0.06 | 0.31 | 0.56 | <.001 | .34 |
|  | Relationship Type | 0.02 | 0.07 | -0.10 | 0.14 | .768 | .02 |
|  | Gender | -0.03 | 0.06 | -0.15 | 0.09 | .680 | .02 |
|  | Visualization X Relationship | -0.08 | 0.06 | -0.20 | 0.04 | .203 | .07 |
|  | Visualization X Gender | 0.03 | 0.06 | -0.09 | 0.15 | .638 | .03 |
|  | Relationship X Gender | 0.15 | 0.06 | 0.03 | 0.28 | .017 | .13 |
|  | Simple effect of recall condition for   men | 0.17 | 0.10 | 0.00 | 0.34 | .070 | .10 |
|  | Simple effect of recall condition for   women | -0.13 | 0.09 | -0.31 | 0.03 | .126 | .08 |
|  | Simple effect of gender for friend   condition | -0.18 | 0.10 | -0.38 | 0.01 | .081 | .09 |
|  | Simple effect of gender for partner   condition | 0.13 | 0.08 | -0.02 | 0.27 | .100 | .09 |
|  | Visualization X Relationship X Gender | -0.09 | 0.06 | -0.21 | 0.03 | .172 | .07 |
|  |  |  |  |  |  |  |  |
| Coherence as Outcome | |  |  |  |  |  |  |
|  | Baseline Coherence | 0.89 | 0.03 | 0.83 | 0.95 | <.001 | .84 |
|  | Visualization Condition | 0.10 | 0.04 | 0.02 | 0.17 | .013 | .13 |
|  | Relationship Type | 0.03 | 0.04 | -0.05 | 0.11 | .443 | .04 |
|  | Gender | 0.00 | 0.04 | -0.08 | 0.07 | .951 | .00 |
|  | Visualization X Relationship | 0.02 | 0.04 | -0.06 | 0.09 | .651 | .02 |
|  | Visualization X Gender | 0.04 | 0.04 | -0.04 | 0.11 | .366 | .05 |
|  | Relationship X Gender | -0.03 | 0.04 | -0.10 | 0.05 | .519 | .03 |
|  | Visualization X Relationship X Gender | -0.01 | 0.04 | -0.08 | 0.07 | .873 | .01 |
|  |  |  |  |  |  |  |  |
| Identification as Outcome | |  |  |  |  |  |  |
|  | Baseline Identification | 0.87 | 0.03 | 0.82 | 0.92 | <.001 | .86 |
|  | Visualization Condition | 0.09 | 0.03 | 0.03 | 0.16 | .005 | .15 |
|  | Relationship Type | 0.00 | 0.03 | -0.07 | 0.07 | .945 | .00 |
|  | Gender | 0.05 | 0.03 | -0.02 | 0.11 | .144 | .08 |
|  | Visualization X Relationship | -0.07 | 0.03 | -0.13 | -0.01 | .024 | .12 |
|  | Visualization X Gender | 0.02 | 0.03 | -0.05 | 0.08 | .621 | .03 |
|  | Relationship X Gender | 0.01 | 0.03 | -0.05 | 0.08 | .669 | .02 |
|  | Visualization X Relationship X Gender | 0.00 | 0.03 | -0.06 | 0.07 | .890 | .01 |

*Note.* Gender was effects-coded as -1 women, +1 men. *LL* = lower limit. *UL* = upper limit.

**Table S7**

*Gender Effects for Study 4 (ANOVA model)*

| Effect | | *df* | *F* | *p* | *n*_p_^2^ |
| --- | --- | --- | --- | --- | --- |
| Change from Pretest to Posttest Understanding | |  |  |  |  |
|  | Visualization Condition (Low vs. High) | 1, 345 | 3.86 | .050 | .011 |
|  | Relationship Type (Friend vs. Partner) | 1, 345 | 9.90 | .002 | .028 |
|  | Gender (Men vs. Women) | 1, 345 | 1.05 | .307 | .003 |
|  | Time (Pretest vs. Posttest) | 1, 345 | 0.06 | .804 | <.001 |
|  | Visualization X Relationship | 1, 345 | 0.08 | .772 | <.001 |
|  | Visualization X Gender | 1, 345 | 0.50 | .479 | .001 |
|  | Relationship X Gender | 1, 345 | 1.99 | .160 | .006 |
|  | Visualization X Time | 1, 345 | 40.73 | <.001 | .106 |
|  | Relationship X Time | 1, 345 | 1.77 | .185 | .005 |
|  | Gender X Time | 1, 345 | 0.99 | .322 | .003 |
|  | Visualization X Relationship X Gender | 1, 345 | 2.92 | .088 | .008 |
|  | Visualization X Relationship X Time | 1, 345 | 2.41 | .121 | .007 |
|  | Visualization X Gender X Time | 1, 345 | 0.02 | .886 | <.001 |
|  | Relationship X Gender X Time | 1, 345 | 3.55 | .060 | .010 |
|  | Visualization X Relationship X Gender X Time | 1, 345 | 0.37 | .545 | .001 |

1. Moreover, the association for understanding was marginally stronger than for caring, *F*(1,111)=3.83, *p*=.053. [↑](#footnote-ref-1)
